# Differential and convergent utilization of autophagy components by positive-strand RNA viruses

**DOI:** 10.1371/journal.pbio.2006926

**Published:** 2019-01-04

**Authors:** Emma Abernathy, Roberto Mateo, Karim Majzoub, Nick van Buuren, Sara W. Bird, Jan E. Carette, Karla Kirkegaard

**Affiliations:** 1 Department of Genetics, Stanford University School of Medicine, Stanford, California, United States of America; 2 Department of Microbiology and Immunology, Stanford University School of Medicine, Stanford, California, United States of America; 3 INSERM U1110, Institute of Viral and Liver Diseases, University of Strasbourg, Strasbourg, France; University of Wisconsin-Madison, United States of America

## Abstract

Many viruses interface with the autophagy pathway, a highly conserved process for recycling cellular components. For three viral infections in which autophagy constituents are proviral (poliovirus, dengue, and Zika), we developed a panel of knockouts (KOs) of autophagy-related genes to test which components of the canonical pathway are utilized. We discovered that each virus uses a distinct set of initiation components; however, all three viruses utilize autophagy-related gene 9 (ATG9), a lipid scavenging protein, and LC3 (light-chain 3), which is involved in membrane curvature. These results show that viruses use noncanonical routes for membrane sculpting and LC3 recruitment. By measuring viral RNA abundance, we also found that poliovirus utilizes these autophagy components for intracellular growth, while dengue and Zika virus only use autophagy components for post-RNA replication processes. Comparing how RNA viruses manipulate the autophagy pathway reveals new noncanonical autophagy routes, explains the exacerbation of disease by starvation, and uncovers common targets for antiviral drugs.

## Author summary

Viruses often co-opt host cellular processes to replicate their genomes and spread to other cells. Many of these cellular pathways provide good targets for antiviral drugs, as they are less likely to develop resistance since they are encoded in the host and not the fast-evolving viral genome. The autophagy pathway is an important stress response pathway that allows cells to recycle cellular components for energy conservation by sequestering cytoplasmic molecules and organelles in double-membraned vesicles (DMVs) and by degrading the contents into reusable elements. Many RNA viruses induce this pathway to provide membrane surfaces for replication and as a source of vesicles for maturation and exit from cells. We developed a panel of CRISPR (Clustered Regularly Interspaced Short Palindromic Repeats) knockout (KO) human cells lacking individual components of the autophagy pathway to assess what aspects of the pathway diverse RNA viruses utilized. We discovered that poliovirus, dengue virus, and Zika virus all use different initiation components of the autophagy pathway but similar downstream components. Additionally, we found that poliovirus uses autophagy components for genome replication, while dengue and Zika viruses use autophagy components for postreplication processes. Ultimately, we uncovered potential drug targets for multiple RNA viruses.

## Introduction

**Fig 1:**
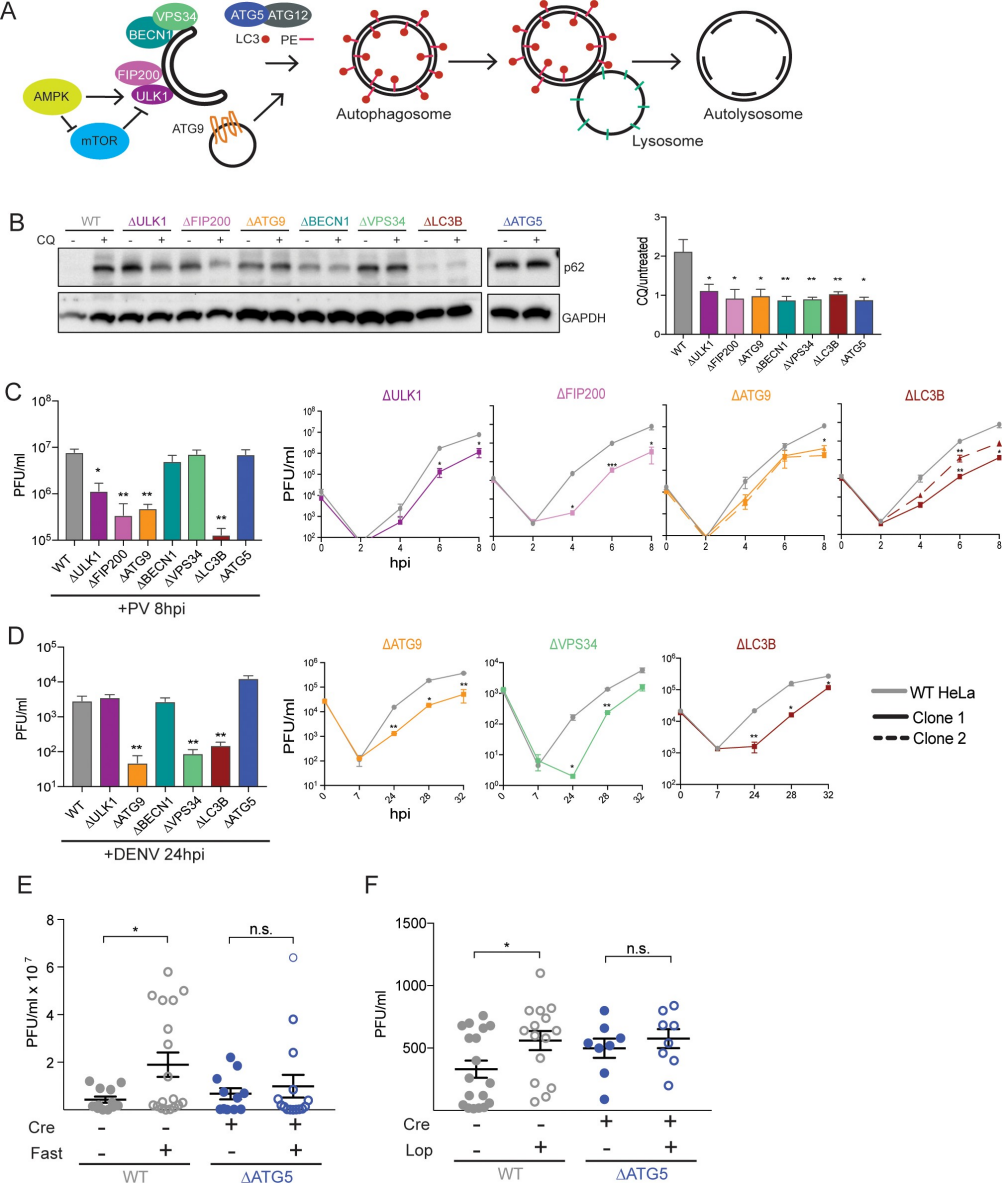
Diverse viruses utilize different components of the autophagy pathway. (A) Schematic of the autophagy pathway and the components targeted in this study for KO by CRISPR-Cas9. (B) Human HeLa cells were left untreated or treated with CQ for 2 hours. Protein lysates were collected and immunoblotted for p62 and GAPDH. Quantification of autophagic flux was done by comparing p62/GAPDH in CQ and untreated samples. (C and D) HeLa cells were infected with PV or DENV at a MOI of 0.1; PFU/cell and cells (PV) or supernatant (DENV) were harvested at indicated times post infection for titration by plaque assay. (E and F) C57BL/6 PVR^+/+^ *Atg5*^fl/fl^ Cre^+/−^ mice were fasted for 48 hours and then infected intramuscularly with PV for E or administered loperamide every 12 hours by i.p. injection during the 4 days of infection for F. Calf muscle tissue was collected and titered by plaque assay at 4 dpi. Open circles denote fasted or drug-treated mice and blue circles indicate Atg5-deficient mice. All data are represented as mean +/− SEM. *Indicates significant *P* value of <0.05, **P value < 0.01, ***P value < 0.001, ****P value > 0.0001 by an unpaired *t* test (C and D) or a Mann–Whitney test (E–F). See also S1 and S2 Figs, S1 and S2 Tables, and S1 Data. Atg5, autophagy-related gene 5; CRISPR, Clustered Regularly Interspaced Short Palindromic Repeats; CQ, chloroquine; DENV, dengue virus; dpi; GAPDH, glyceraldehyde 3-phosphate dehydrogenase; HeLa, human epithelial-derived cell line; i.p., intraperitoneal; KO, knockout; MOI, multiplicity of infection; PFU, plaque-forming units; PV, poliovirus; PVR, poliovirus receptor.

Manipulation of the autophagy pathway is a burgeoning field of research, providing many potential targets for antiviral, anticancer, and neuro-preservation therapies. The autophagy pathway is a highly conserved cellular response that is induced upon nutrient deprivation, other stresses, and developmental cues. Canonical autophagy (“self-eating”) proceeds through a series of distinct steps that nucleate and expand membranous structures, termed autophagosomes, that enclose cytoplasmic contents [1] (Fig 1A). The resulting double-membraned vesicles (DMVs) then fuse with lysosomes, in which hydrolases promote degradation of the cytoplasmic contents for reuse by the cell. The autophagy pathway utilizes large amount of lipids to accomplish the formation of autophagosomes. The origins of these membranes are debated but are likely to derive both from the endoplasmic reticulum (ER) and lipids scavenged from a variety of membranes throughout the cell [2–6].

There are over 30 autophagy-related genes (ATGs) that were first identified in yeast, most of which have mammalian homologs [1]. Autophagy gene products take part in several distinct complexes (Fig 1A). In human cells, the specific kinase activity of the complex that contains Unc-like autophagy-activating kinase (ULK1), PTK2/FAK family interacting protein of 200 kDa (FIP200), ATG13, and ATG101 complex is normally repressed by mammalian target of rapamycin (mTOR) under basal conditions. Upon mTOR activation, ULK1/FIP200/ATG101 is activated, thus activating the downstream autophagy pathway [7]. mTOR repression of the ULK1 complex activity can be bypassed by another kinase, AMP-activated protein kinase (AMPK). Thus, ULK1 activation can be achieved by two different routes of autophagy induction [8]. This complex is essential for the downstream formation of autophagosomes via the kinase activity of ULK1 [9]. The VPS34/ beclin-1(BECN1)/ATG14L complex, also essential for the initiation and maturation of autophagosomes, relies on ULK1 kinase activity, with BECN1 serving as a direct substrate [10]. The kinase activity of VPS34 is in turn required for the production of phosphatidylinositol lipids needed to recruit downstream autophagy proteins [11,12]. The activation of ATG9, the only known intrinsically membrane-bound protein in the pathway, also requires ULK1. ATG9 is important for scavenging lipids from various sites throughout the cell and trafficking them back to growing autophagosomes [13–17]. These events lead to ATG5-mediated covalent attachment of phosphatidylethanolamine (PE) to light-chain 3 (LC3) [18], the most characteristic component of immature and mature autophagosomes. Membrane-associated LC3 is needed for recruitment of cargo, stabilization of negative curvature, closure of the autophagosome, and fusion with lysosomes [19–22]. While each of these proteins is considered essential for the canonical autophagy pathway, several instances of mammalian autophagy that lack one or more of these components have been reported, including ULK1-independent, BECN1-independent, and ATG5-independent formation of DMVs and degradation of cytoplasmic contents [23–25]. Such processes are termed “noncanonical” autophagy. This also includes secretory autophagy, in which cytoplasmic components are released undegraded into the extracellular milieu [26].

Many viruses interact with the autophagy pathway, which can play both antiviral and proviral roles, sometimes even during the same viral infections. Antiviral activities of autophagy include xenophagy, in which intracellular viruses are targeted for degradation as well as the triggering and facilitation of immune responses [27]. As a testament to the power of autophagy as an arm of innate immunity, many successful viruses such as herpesviruses [28], Sindbis virus [29], and vesicular stomatitis virus [30] have evolved strategies to block the induction of the autophagy pathway. On the other hand, utilization of components of the autophagy pathway by many viruses serves proviral functions. The unique ability of the autophagy pathway to generate membranous structures de novo and to allow or disallow their acidification may be desirable features for any virus that survives by manipulating intracellular membranes. All positive-strand RNA viruses associate with cytoplasmic membranes to replicate their genomes. Virally associated membranes come from a variety of cellular compartments, including ER, Golgi, mitochondria, and lysosomes [31–34]. Many viral proteins are membrane-bound, and RNA amplification occurs on the topologically cytoplasmic surfaces of vesicles. Viruses such as dengue, for instance, induce ER-membrane invaginations to form isolated pockets in which viral proteins congregate for RNA replication outside the reach of cellular antiviral factors.

For poliovirus (PV), dengue virus (DENV), and Zika virus (ZIKV), proviral roles of the autophagy pathway and its components have been documented. PV and other picornaviruses have been shown to induce the formation of autophagosome-like membranes for purposes of RNA replication, virion maturation, and nonlytic viral spread [34–40]. Coxsackievirus B3 (CVB3) has also been shown to use the autophagy pathway for viral spread and to induce the formation of DMVs [37,41]. DENV has been shown to induce the proliferation of LC3-containing membranes [31,42–44]. Inhibiting PI 3-kinases, including VPS34, with 3-methyladenine (3-MA) decreases DENV yield [45], and a specific autophagy inhibitor, spautin-1, deranges the maturation of DENV particles [46]. ZIKV has also been shown to induce the formation of LC3-containing membranes [47,48]. Furthermore, one study suggests that noncanonical secretory autophagy may contribute to the spread of ZIKV [49], as it does with PV and CVB3.

To determine which components of the autophagy pathway are used by these RNA viruses and whether there are any shared principles, we generated a panel of CRISPR (Clustered Regularly Interspaced Short Palindromic Repeats)-Cas9 knockout (KO) human epithelial-derived cell lines (HeLa). Previously, most research investigating viruses and cellular autophagy has involved targeting single genes genetically or pharmaceutically. This does not take into account the potential utilization of noncanonical autophagy pathways or gain-of-function effects of drugs [50]. We found that PV, DENV, and ZIKV all utilize multiple components of the autophagy pathway while bypassing others and that each virus uses a unique set of initiation components. A common feature is that all of the tested viruses require the LC3 protein but bypass its canonical cellular lipidation process, using other means to recruit LC3 to virally induced membranes. This study highlights the importance of assessing the full autophagy pathway when seeking to understand how pathogens manipulate this pathway for purposes of genome replication and spread, as well as potential common drug targets.

## Results

### Diverse viruses utilize different components of the autophagy pathway

To provide an in-depth characterization of which autophagy components are utilized by the three different viruses, we targeted either one or two genes from distinct complexes required for canonical autophagy (Fig 1A). Sequencing confirmed the genomic ablation of the targeted genes in Δ*FIP200*, Δ*ATG9*, Δ*BECN1*, Δ*VPS34*, Δ*LC3*, and Δ*ATG5* (S1A Fig). For GC-rich *ULK1*, specific antibodies confirmed a functional loss of protein, as they did for the targeted gene products in all the CRISPR-Cas9 KO cell lines (S1B Fig). To test whether canonical autophagy was inactivated in these KO cell lines, the accumulation of p62/SQSTM (p62), which is degraded in wild-type (WT) cells via constitutive autophagy, was monitored (Fig 1B). Accumulated p62 was present at low abundance in the parental HeLa cells (WT) and increased in cells treated with chloroquine (CQ) to prevent the formation of autolysosomes and thus the degradation of autophagosomal contents. All KO cell lines showed enhanced constitutive p62 accumulation, whether or not they were treated with CQ, indicating that the canonical basal autophagy pathway was successfully inhibited (Fig 1B). Additionally, to confirm that the canonical, starvation-induced autophagy pathway was obstructed in all of the KO cell lines, we quantified the presence of endogenous LC3 puncta upon starvation. WT cells displayed significantly more puncta upon starvation than any of the KO lines (S1C Fig).

We then took advantage of these generated KO cell lines to determine which components of the autophagy pathway affect viral growth for a picornavirus (PV) and a flavivirus (DENV). PV is a nonenveloped virus that matures intracellularly. The intracellular amplification of PV in WT and KO cell lines was monitored in a single infectious cycle, which comprises cell entry, translation, RNA replication, and packaging but not cell-to-cell spread. As shown in Fig 1C, cells ablated for *ULK1*, *FIP200*, *ATG9*, and *LC3B* had significant viral growth defects at this time point. However, viral titers in cells ablated for *BECN1*, *VPS34*, and *ATG5* were indistinguishable from WT. More detailed time courses of a single PV infectious cycle confirmed the reduction in virus yield in Δ*ULK1*, Δ*FIP200*, Δ*ATG9*, and Δ*LC3* cells (Fig 1C). The fact that ATG5 is not required is particularly curious, given that viral growth was drastically reduced in the Δ*LC3B* line (Fig 1C). These observations argue that the virus still utilizes the LC3 protein, though perhaps not its lipidated form. We confirmed that adding back autophagy components to our KO cell lines restored viral titers to WT levels for ULK1 and FIP200 (S2A Fig). Additionally, adding back a kinase dead ULK1 mutant (K46I) reduced viral yield in WT cells, consistent with its previously reported dominant negative effects on the autophagy pathway [51]. ULK1–K46I also failed to rescue the viral defect in ULK1 KO cells (S2A Fig).

DENV is an enveloped virus that matures upon release from cells after budding into the ER and traversing the canonical downstream secretion pathway. We monitored a single cycle of DENV growth by quantifying the release of infectious virus into the extracellular medium. All CRISPR-Cas9 KO cells were infected with DENV for a single 24-hour replication cycle at a low multiplicity of infection (MOI) (Fig 1D). We found that DENV does not require the same initiation complex as PV, as viral titers were unaffected in Δ*ULK1* cells. We additionally tested whether a double knock down of ULK1 and ULK2 affected DENV growth, given that ULK1 and ULK2 can be redundant in some cell types [9,52]. We saw no defect in DENV titers when small interfering RNAs (siRNAs) against ULK2 were used in the Δ*ULK1* cell line or when ULK1 and ULK2 siRNAs were used in the Δ*FIP200* cell line (S2B Fig), suggesting that DENV does not require this complex. Similar to PV, DENV titers were reduced in Δ*ATG9* cells, perhaps highlighting a common need for lipid scavenging in HeLa cells. DENV utilized VPS34 but not BECN1, both part of the same complex, arguing that DENV might specifically require VPS34 but not the rest of the complex. Additionally, adding back VPS34 to the Δ*VPS34* cell line restored DENV titers to WT levels (S2C Fig). Downstream in the autophagy pathway, DENV utilized LC3 but not ATG5, similarly to PV.

DENV and PV infection in cell culture benefit from the induction of the canonical autophagy pathway when treated with autophagy inducers such as rapamycin [46] or loperamide [39]. Recently, we showed that induction of the autophagy pathway by starvation led to an enhancement of PV amplification that was dependent on the canonical pathway, as Δ*ATG5* and Δ*BECN1* cell lines failed to show enhanced viral infection upon starvation [53]. To test whether in vivo PV infection is also enhanced by starvation as well as whether this exacerbation depends on the canonical autophagy pathway, we bred C57BL/6 mice that were homozygous for a transgene expressing the poliovirus receptor (PVR) [54] as well as the *Atg5* gene flanked by Lox sites, the targets of Cre recombinase. These mice were bred either to express or not express Cre recombinase under the control of a tamoxifen-inducible promoter [55]. The conditional deletion of floxed *Atg5* upon tamoxifen treatment was confirmed by quantitative PCR (qPCR) of a DNA junction expected to be generated during the Cre-mediated excision (S2D Fig). The decrease of Atg5 function in the Cre-expressing mice was confirmed at the protein level by the large increase in abundance of LC3-I, which normally would be converted to LC3-II by Atg5 (S2E Fig). Although a small amount of residual LC3-II remained in the Cre-expressing mice, this is typical of the nonabsolute nature of “floxed” gene removal by Cre recombinase [55]. Mice were fasted for 48 hours before intramuscular infection with PV. Fasted mice showed increased PV titers in mice with an intact Atg5 gene (Fig 1E). However, in the absence of Atg5, the enhanced viral titer seen with fasting was not observed (Fig 1E). Additionally, we treated mice with loperamide to induce the autophagy pathway by a different mechanism [56,57] and found a similar response of enhanced viral titers in WT mice treated with the drug but no difference between untreated and drug-treated mice lacking Atg5 (Fig 1F). These data argue that the exacerbation of viral infection by starvation or loperamide-induced autophagy requires ATG5 and most likely the canonical pathway, even though poliovirus growth per se uses only portions of this pathway. We speculate that preinduction of the canonical autophagy pathway increases the concentrations of proteins or lipids that facilitate subsequent viral infection.

### PV uses components of the autophagy pathway for early replicative events, while DENV and ZIKV use it for post-RNA replication processes

**Fig 2:**
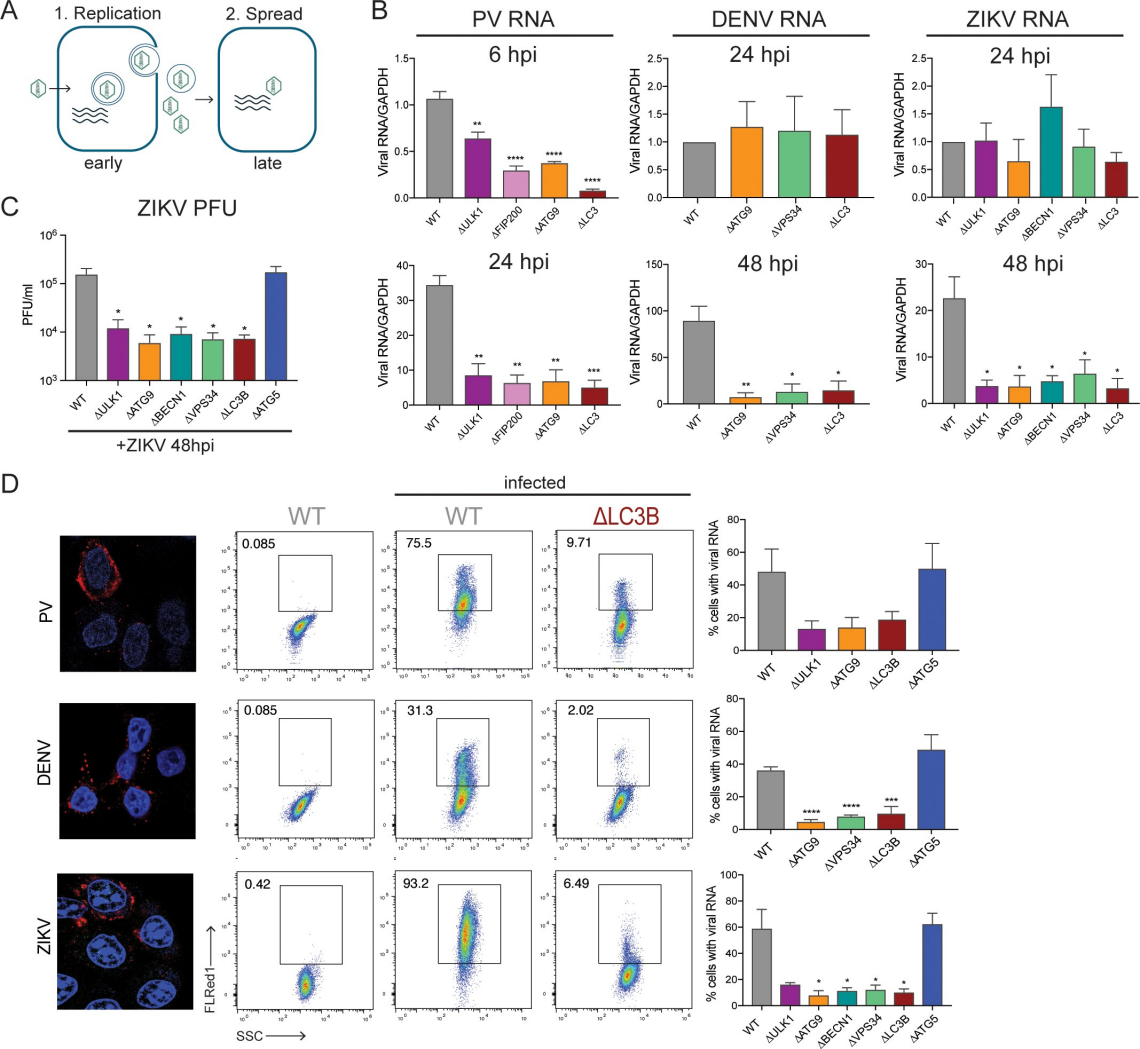
PV uses components of the autophagy pathway for early replicative events, while DENV and ZIKV use it for post-RNA replication processes. (A) Schematic of viral replication and spread. (B) HeLa cells were infected with PV, DENV, or ZIKV at an MOI of 0.1 PFU/cell, and RNA was harvested at 6, 24, or 48 hpi. Viral RNA abundance was measured by RT-qPCR and normalized to GAPDH. (C) HeLa cells were infected with ZIKV at an MOI of 0.1 PFU/cell, and supernatant was harvested at 48 hpi. Viral titers were determined by plaque assay. (D) WT HeLa cells were infected with PV, DENV, or ZIKV at an MOI of 1 PFU/cell for 24 hours. Cells were fixed and stained with viral-specific fluorescent probes and visualized by confocal microscopy. For flow cytometry, HeLa cells were infected at an MOI of 0.1 PFU/cell for 24 hours (PV) or 48 hours (DENV, ZIKV). Cells were fixed and stained with viral-specific probes. Percent infection was determined by gating on the uninfected controls. Representative FACS plots are shown. All data are represented as mean +/− SEM. *Indicates significant *P* value of <0.05, **P value < 0.01, ***P value < 0.001, ****P value > 0.0001 by an unpaired *t* test. See also S3 Fig and S2 Data. DENV, dengue virus; GAPDH, glyceraldehyde 3-phosphate dehydrogenase; HeLa, human epithelial-derived cell line; hpi, hours post infection; MOI, multiplicity of infection; PFU, plaque-forming units; PV, poliovirus; RT-qPCR, reverse transcription quantitative PCR; WT, wild-type; ZIKV, Zika virus.

Previous experiments have argued that in the absence of cellular autophagy or its components, intracellular RNA amplification as well as later steps are defective in PV-infected cells [36,39]. On the other hand, DENV-infected cells lacking an intact autophagy pathway synthesize viral RNA, but virion maturation is defective [46]. To distinguish between amplification in the first round of infection and subsequent spread, intracellular RNA accumulation was monitored by reverse transcription quantitative PCR (RT-qPCR) at early and late time points (Fig 2A). Even in the first cycle of PV infection in the KO cell lines, the decline in RNA accumulation was significant in Δ*ULK1*, Δ*FIP200*, Δ*ATG9*, and Δ*LC3* cells, and this effect continued upon subsequent rounds (Fig 2B). To test whether viral entry was affected in the KO cell lines, we performed an entry assay. Virus was allowed to infect cells for 30 minutes, after which virions that had not entered were stripped from the cell surface with an acid wash. RT-qPCR analysis of PV RNA that successfully entered the cell showed no difference between WT and KO cell lines (S3A Fig), indicating that the RNA accumulation defect lies downstream of entry. Intracellular accumulation of viral protein 2C was reduced in the KO cell lines shown (S3B Fig), consistent with a defect in translation, RNA replication, or both. Similarly, transfection of a PV replicon that expressed firefly luciferase showed reduced protein accumulation in the KO cell lines (S3C Fig). To distinguish between defects in viral translation and RNA replication, we monitored translation at a 2-hour time point in the absence and presence of guanidine, a specific inhibitor of RNA replication. Only WT cells differed in their accumulation of luciferase in the absence or presence of guanidine (S3D Fig). Therefore, it is RNA replication that is specifically inhibited in the KO lines.

To determine whether autophagy components influence DENV translation and RNA synthesis, viral packaging and spread, or both, viral RNA synthesis was monitored after single (24 hpi [hours post infection]) and multiple (48 hpi) rounds of infection. In the absence of *ATG9*, *VPS34*, or *LC3*, no reduction in DENV RNA was observed in the first cycle (Fig 2B), even though the production of infectious virus was significantly reduced at this time point (Fig 1D). This is consistent with previous observations that in the presence of autophagy inhibitor spautin-1, packaged virions were defective but RNA accumulation was not [46]. Upon additional rounds of infection, the accumulation of DENV viral RNA was greatly reduced (Fig 2B), consistent with the poor infectivity of virions from the first round. In accordance with these data, we observed similar amounts of viral protein accumulation at early time points in WT and Δ*ATG9* cells but reduced accumulation at later time points (S3E Fig). We conclude that in the absence of a noncanonical autophagy pathway, initial DENV translation and RNA synthesis are not affected, but infection of subsequent rounds is greatly reduced as a result of defective assembly, maturation, or egress.

ZIKV is a flavivirus that is similar to DENV but infects a larger number of cell types in humans [47]. ZIKV RNA was monitored during infection, and like DENV, viral RNA abundance remained similar to WT in the first infectious cycle (24 hpi). However, after additional infectious cycles, both RNA (Fig 2B) and virus production (Fig 2C) were greatly reduced. These data argue that for ZIKV, like DENV, autophagy components are only necessary for a postreplication process such as packaging or particle maturation. However, unlike either PV or DENV, the accumulation of infectious ZIKV was reduced in the entire CRISPR-Cas9 KO panel except the Δ*ATG5* line (Fig 2C). This argues that ZIKV utilizes both upstream initiation complexes ULK1/FIP200 and BECN1/VPS34, perhaps feeding into many aspects of the canonical autophagy pathway. Nonetheless, the fact that all three viruses bypass the need for ATG5-mediated lipidation of LC3B highlights a shared strategy.

To monitor the accumulation of viral RNAs on a single-cell basis, we measured the number of infected cells during multiple cycles of infection by flow cytometry. Virus-specific probes were designed to hybridize to each positive-strand viral genome. These probes can subsequently be bound by branched DNA structures, with each DNA “tree” containing thousands of fluorophores as a means to amplify the signal, which can allow the detection of single RNA molecules [58,59]. The low background for these probes from uninfected cells was validated in WT HeLa cells by confocal microscopy (Fig 2D). These probes were then used to determine the percentage of cells positive for viral RNA by flow cytometry, using a low MOI (0.1 plaque forming units [PFU]/cell) and multiple cycles of infection. In the cell lines that showed significant defects in growth for each virus, fewer cells were positive for PV, DENV, or ZIKV viral RNA (Fig 2D).

### Differential utilization of upstream autophagy components by RNA viruses

**Fig 3:**
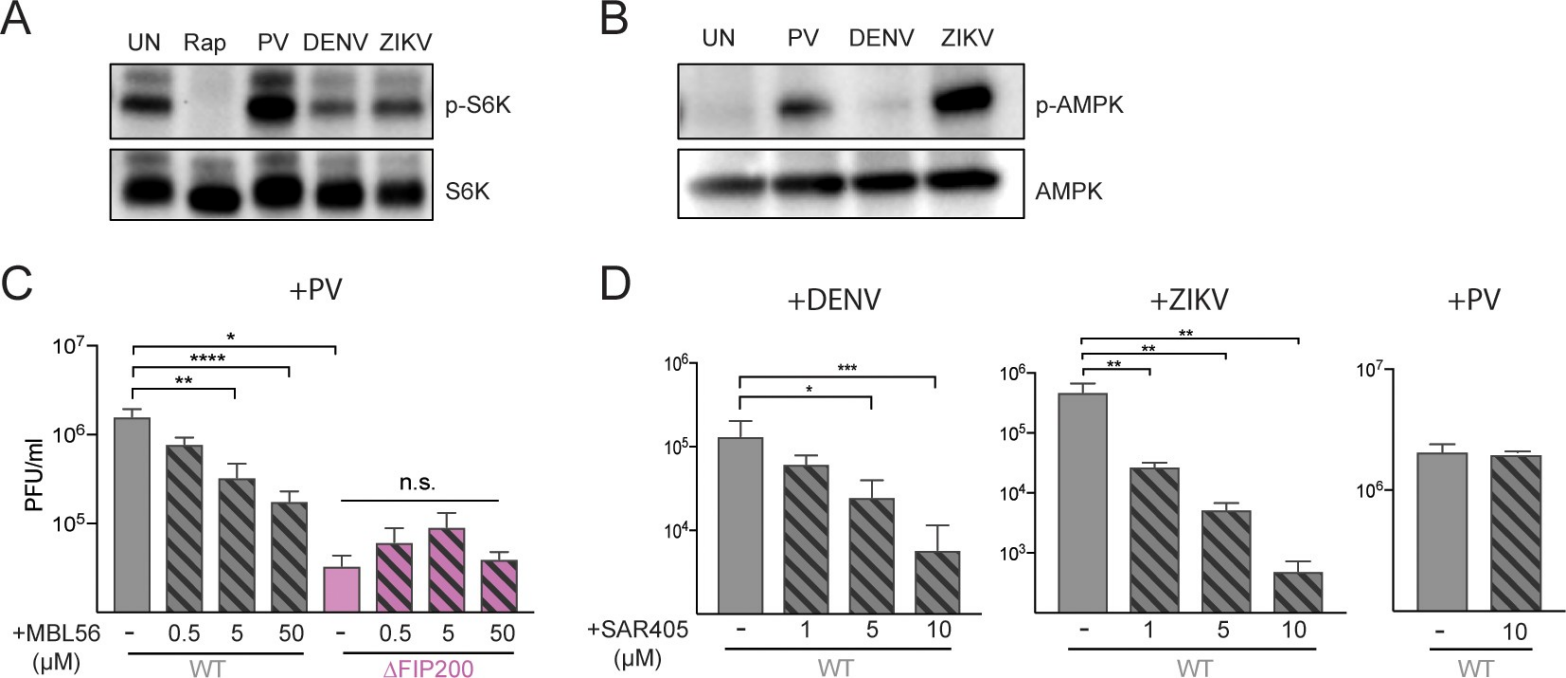
Differential utilization of upstream autophagy components by RNA viruses. (A and B) Cells were treated with rapamycin or infected with PV (6 hpi), DENV (24 hpi), or ZIKV (24 hpi). Cell lysates were harvested and immunoblotted with phospho-S6K or phospho-AMPK antibodies. (C) Cells were treated with 0.5, 5, or 50 μM of MBL56 and infected with PV at an MOI of 0.1 PFU/cell for 6 hours. (D) WT cells were pretreated with 1, 5, or 10 μM of SAR405 and infected with DENV or ZIKV at an MOI of 0.1 PFU/cell for 24 hours (DENV) or 48 hours (ZIKV). WT cells were pretreated with 10 μM of SAR405 and infected with PV at an MOI of 0.1 PFU/cell for 6 hours. All data are represented as mean +/− SEM. *Indicates significant *P* value of <0.05, **P value < 0.01, ***P value < 0.001, ****P value > 0.0001 by an unpaired *t* test. See also S3 Data. AMPK, AMP-activated protein kinase; DENV, dengue virus; hpi, hours post infection; MOI, multiplicity of infection; PFU, plaque-forming units; PV, poliovirus; WT, wild-type; ZIKV, Zika virus.

Autophagy can be induced by two different arms of upstream signaling: mTOR inactivation, leading to dephosphorylation of ULK1 and thus its activation, or AMPK activation, leading to a distinct phosphorylation of ULK1 in the absence of mTOR repression [8,60,61]. mTOR typically responds to nutrient signals while AMPK responds to the energy status of the cell. Other viruses have been shown previously to activate the autophagy pathway via AMPK activation [62]. The differential utilization of upstream autophagy components by PV, DENV, and ZIKV could result from the activation of different upstream signaling cascades. To test this possibility, we looked at the phosphorylation status of S6K, which is phosphorylated when mTOR is active and autophagy is repressed [63]. We saw that while rapamycin treatment led to the loss of S6K phosphorylation, PV, DENV, and ZIKV infection did not alter its phosphorylation status (Fig 3A). This suggests that the activation of autophagy during viral infection is independent of mTOR inactivation and thus, by definition, noncanonical. We next looked at AMPK phosphorylation status and discovered that PV and ZIKV led to AMPK phosphorylation and thus activation but not DENV (Fig 3B). Since AMPK is known to directly activate ULK1, these data support the idea that PV and ZIKV utilize the ULK1 complex in activating the autophagy pathway, while DENV bypasses both mTOR and AMPK, perhaps avoiding ULK1 activation entirely.

To determine whether ULK1 and FIP200 function in the same way in stimulating PV growth as they do in canonical autophagy, we used a small molecule inhibitor of ULK1 (MBL56), which specifically blocks its kinase activity [64]. In WT HeLa cells, addition of MBL56 decreased viral titers after 6 hours of infection in a dose-dependent manner (Fig 3C). However, MBL56 had no additional effect on viral titers in infected Δ*FIP200*, arguing that ULK1 and FIP200 function together just as they do in canonical autophagy.

DENV bypasses the upstream ULK1 complex and instead requires VPS34. To determine if DENV requires a catalytically functional VPS34 protein, we used VPS34-specific inhibitor SAR405 [65]. Addition of SAR405 led to reduced DENV titers at 24 hpi in a dose-dependent manner (Fig 3D). We see reduced ZIKV titers with addition of SAR405 as well, showing that ZIKV also requires a catalytically functional VPS34 (Fig 3D). Interestingly, we did not see any reduction in PV titers with addition of SAR405 (Fig 3D), consistent with the previous conclusion that PV does not require VPS34 activity. Therefore, PV and ZIKV induce the autophagy pathway upstream of AMPK, while DENV uses an alternate route to induce autophagy pathway, perhaps through the direct activation of VPS34.

### Virally induced membrane rearrangements are altered in cells that lack individual autophagy components

**Fig 4:**
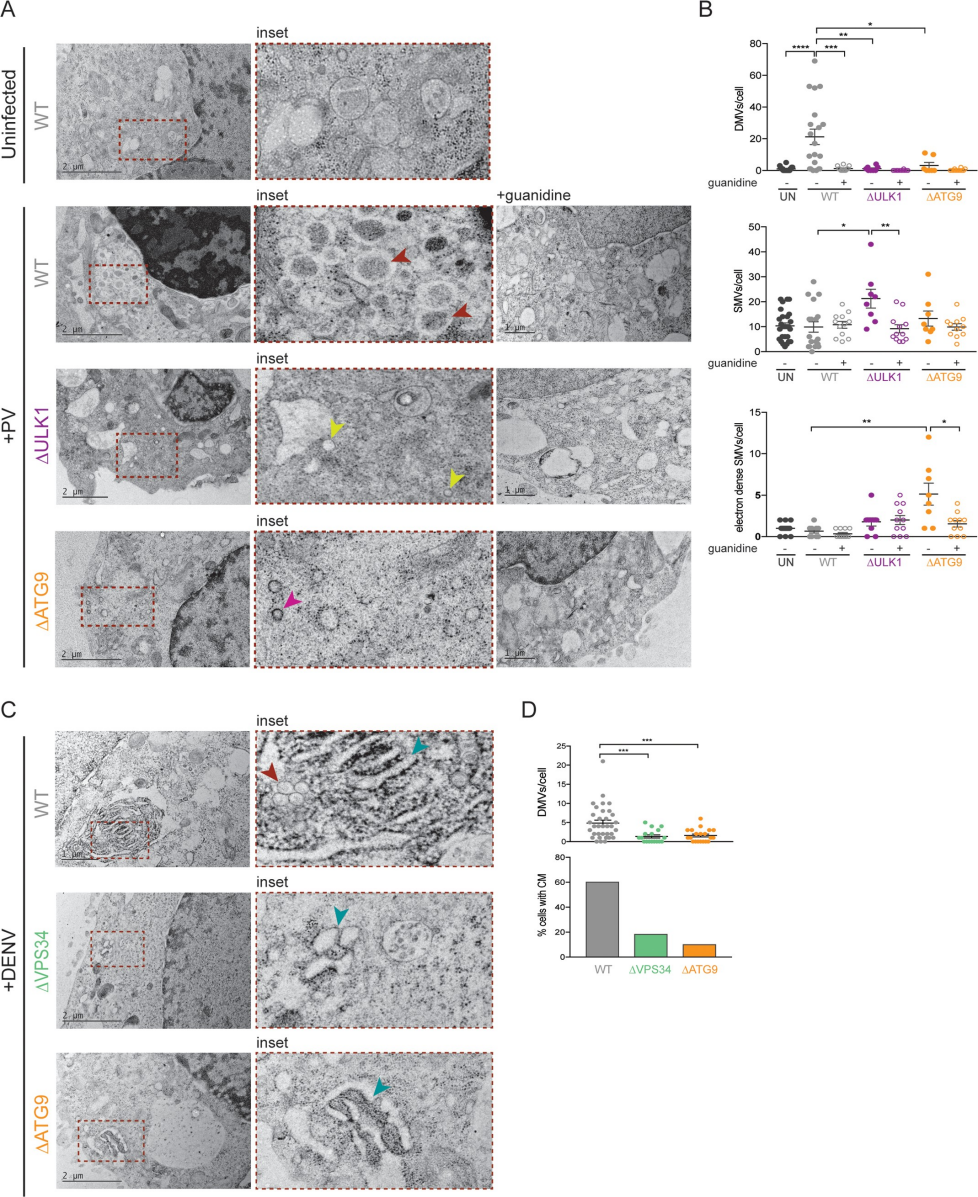
Virally induced membrane rearrangements are altered in cells that lack individual autophagy components. (A and B) HeLa cells were infected with PV or DENV at an MOI of 10 PFU/cell. Indicated cells were treated with 2 mM guanidine during the infection period. Cells were fixed at 6 hpi (PV) or 24 hpi (DENV) and subjected to high-pressure freezing and freeze substitution. Images were collected on a TEM microscope. Representative images are shown. Red arrowheads indicate DMVs, yellow arrowheads indicate SMVs, pink arrowheads indicate electron-dense SMVs, blue arrowheads indicate CM. Quantification of cellular structures was done on blinded images, and >10 cells per condition were counted. All data are represented as mean +/− SEM. *Indicates significant *P* value of <0.05, **P value < 0.01, ***P value < 0.001, ****P value > 0.0001 by a Mann–Whitney test. See also S4 Data. CM, convoluted membranes; DENV, dengue virus; DMV, double-membraned vesicles; HeLa, human epithelial-derived cell line; hpi, hours post infection; MOI, multiplicity of infection; PFU, plaque-forming units; PV, poliovirus; SMV, single-membraned vesicles.

To test whether the extensive membrane rearrangements that occur in cells infected with PV and DENV are altered in cell lines lacking particular autophagy components that affected viral growth, we studied the ultrastructure of infected WT and KO cells. WT, Δ*ULK1*, and Δ*ATG9* cells were infected with PV at a high MOI (10 PFU/cell) for 6 hours, followed by high-pressure freezing and freeze substitution to preserve membrane structures. Images were visualized by electron microscopy (EM) (Fig 4A). PV-infected WT cells showed the characteristic DMVs shown previously to be induced either by infection or coexpression of PV proteins 2BC and 3A [34,35]. These DMV structures were not present when cells were treated with guanidine, a potent inhibitor of viral replication (Fig 4A and 4B). In Δ*ULK1* and Δ*ATG9* cells, qualitatively different membrane rearrangements were observed. Infection of ΔULK1 cells led to an increase in single-membraned vesicles, while infection of Δ*ATG9* cells led to an increased number of electron dense single-membraned vesicles, both of which diminished when cells were treated with guanidine (Fig 4A and 4B). Single-membraned vesicles can be the precursors of DMVs in canonical autophagy; this may also be the case in PV-infected cells [66]. Therefore, the single-membraned vesicles observed in Δ*ULK1* and Δ*ATG9* cells could represent precursors or off-pathway structures that accumulate during arrest of the functions of the ULK1 complex. The presence of such precursors could also be increased by the lower viral protein abundance in these KO cell lines (S3B Fig). No alterations in membrane structures were seen in any cells in the presence of guanidine, arguing that translation from input RNA is not sufficient to induce these changes.

DENV growth does not use the canonical ULK1 complex but instead requires both VPS34 and ATG9 (Fig 1D). To identify any alteration in membrane structure under these conditions, we visualized DENV-infected WT, Δ*VPS34*, and Δ*ATG9* cells by EM. Typically, during a DENV infection, DMVs and large convoluted membrane structures thought to be extensions of the ER are formed by 24 hpi (Fig 4C) [67]. These structures were notably absent in Δ*VPS34* and Δ*ATG9* cells (Fig 4C and 4D), even though comparable amounts of viral RNA and protein were observed in these cell lines at this time point (Fig 2B and S3E Fig), arguing that VPS34 and ATG9 play crucial roles in the architecture of these membrane structures.

### LC3 is recruited to membranes independent of lipidation during viral infection

**Fig 5:**
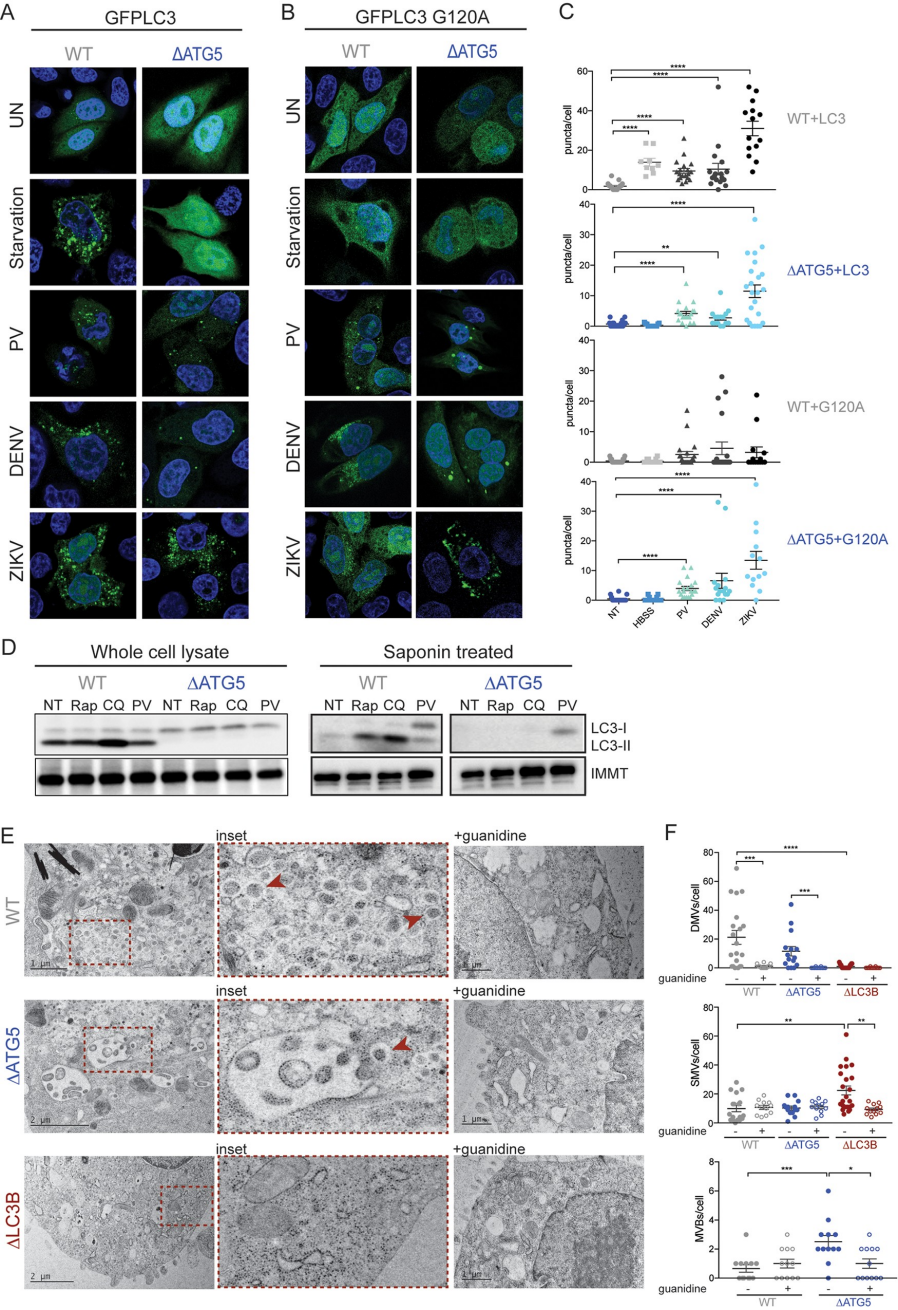
LC3 is recruited to membranes independent of lipidation during viral infection. (A and B) HeLa cells were transfected with GFP–LC3 or GFP–LC3–G120A for 48 hours. Cells were either starved for 2 hours or infected at an MOI of 10 PFU/cell with PV (6 hours), DENV or ZIKV (24 hours) and fixed for visualization by confocal microscopy. (C) Puncta per cell were counted for each condition; *n* = >10 cells. (D) HeLa cells were treated with Rap, CQ, or infected with PV at an MOI of 10 PFU/ml for 6 hours. Lysates were harvested with or without saponin and run on an SDS PAGE gel. Immunoblots were stained for LC3 and the membrane-associated IMMT. (E) HeLa cells were infected with PV at an MOI of 10 PFU/cell and subjected to high-pressure freezing and freeze substitution for visualization on a TEM microscope. Representative images are shown. Red arrowheads indicated DMVs. (F) Cell structures were quantified on blinded images; *n* = >15 cells. All data are represented as mean +/− SEM. *Indicates significant *P* value of <0.05, **P value < 0.01, ***P value < 0.001, ****P value > 0.0001 by a Mann–Whitney test. See also S5 Data. CQ, chloroquine; DENV, dengue virus; DMV, double-membraned vesicle; GFP, green fluorescent protein; HeLa, human epithelial-derived cell line; IMMT, inner membrane mitochondrial protein; LC3, light-chain 3; MOI, multiplicity of infection; PFU, plaque-forming units; PV, poliovirus; Rap, rapamycin.

ATG5 is often considered the hallmark of canonical autophagy due to its essential function in the lipidation of LC3. Considering that we observed no defects in the growth of PV, DENV, or ZIKV in Δ*ATG5* cells but significant dependence on LC3, we were curious about the mechanism of LC3 utilization during these viral infections. To observe the membrane-associated sequestration of LC3 during canonical autophagy and viral infection, we monitored the relocalization of a plasmid expressing a green fluorescent protein (GFP)–LC3 fusion protein [20,68]. Under normal conditions, GFP–LC3 is dispersed throughout the cytoplasm, as shown in the top panel of Fig 5A. Upon induction of the canonical autophagy pathway, GFP–LC3 forms distinct puncta that represent membrane-bound, lipidated LC3. Under starvation conditions, GFP–LC3 puncta form in WT cells but not in Δ*ATG5* cells. Infection with PV, DENV, or ZIKV also led to the formation of GFP–LC3 puncta in both the presence and absence of ATG5 (Fig 5A and 5C).

To test further whether the induction of LC3 puncta by PV, DENV, and ZIKV was truly independent of LC3 lipidation, we expressed a version of GFP–LC3 that contained a G120A mutation, which lacks the essential glycine residue needed for the attachment of PE [69]. As expected, starvation did not induce the formation of GFP–LC3–G120A puncta in either the presence or absence of ATG5 (Fig 5B, upper panels). The fact that very few GFP–LC3–G120A puncta were observed in WT cells might indicate that the virus preferentially induces puncta formation with lipidated LC3, which is still present as untagged endogenous LC3. However, GFP–LC3–G120A puncta formation was observed in Δ*ATG5* cells upon infection with PV, DENV, or ZIKV (Fig 5B and 5C). These results suggest that all three viruses can either recruit unlipidated LC3 to membranes or can lead to lipidation of LC3 independently of ATG5.

To determine whether or not LC3 was lipidated during infection, we enriched for membrane-associated proteins and determined the lipidation status of LC3 by immunoblot. Cells were treated with rapamycin, CQ, or infected with PV. As expected, no LC3-II was observed in ΔATG5 cells, and treatment with CQ increased LC3-II abundance in WT but not ΔATG5 cells when whole-cell lysates were tested (Fig 5D). Proteins most tightly associated with membranes can be enriched by treatment of extracts with saponin [70]. LC3-I was retained in the membrane fraction in PV-infected cells in both WT and Δ*ATG5* cells (Fig 5D). Thus, PV is capable of recruiting LC3-I to membranes in the absence of lipidation and the canonical lipidation machinery. In agreement with the above data, we observed by EM that PV infection still leads to the formation of DMVs in Δ*ATG5* cells (Fig 5E and 5F) and that these structures are dependent on active viral replication since they are absent in cells treated with guanidine. However, no DMVs were seen upon PV infection of Δ*LC3B* cells, but an increase in single-membraned vesicles was observed. Therefore, PV is capable of forming DMVs without lipidated LC3 but still appears to require the LC3 protein itself. Interestingly, PV infection of Δ*ATG5* cells resulted in the formation of many large vesicles that resemble multivesicular bodies (MVBs) (Fig 5E and 5F). These might represent an altered curvature capacity of unlipidated LC3 alone [22], as compared to the increased curvature in PV-infected WT cells, which contain both LC3-I and LC3-II (Fig 5D).

### Viral proteins bind LC3

**Fig 6:**
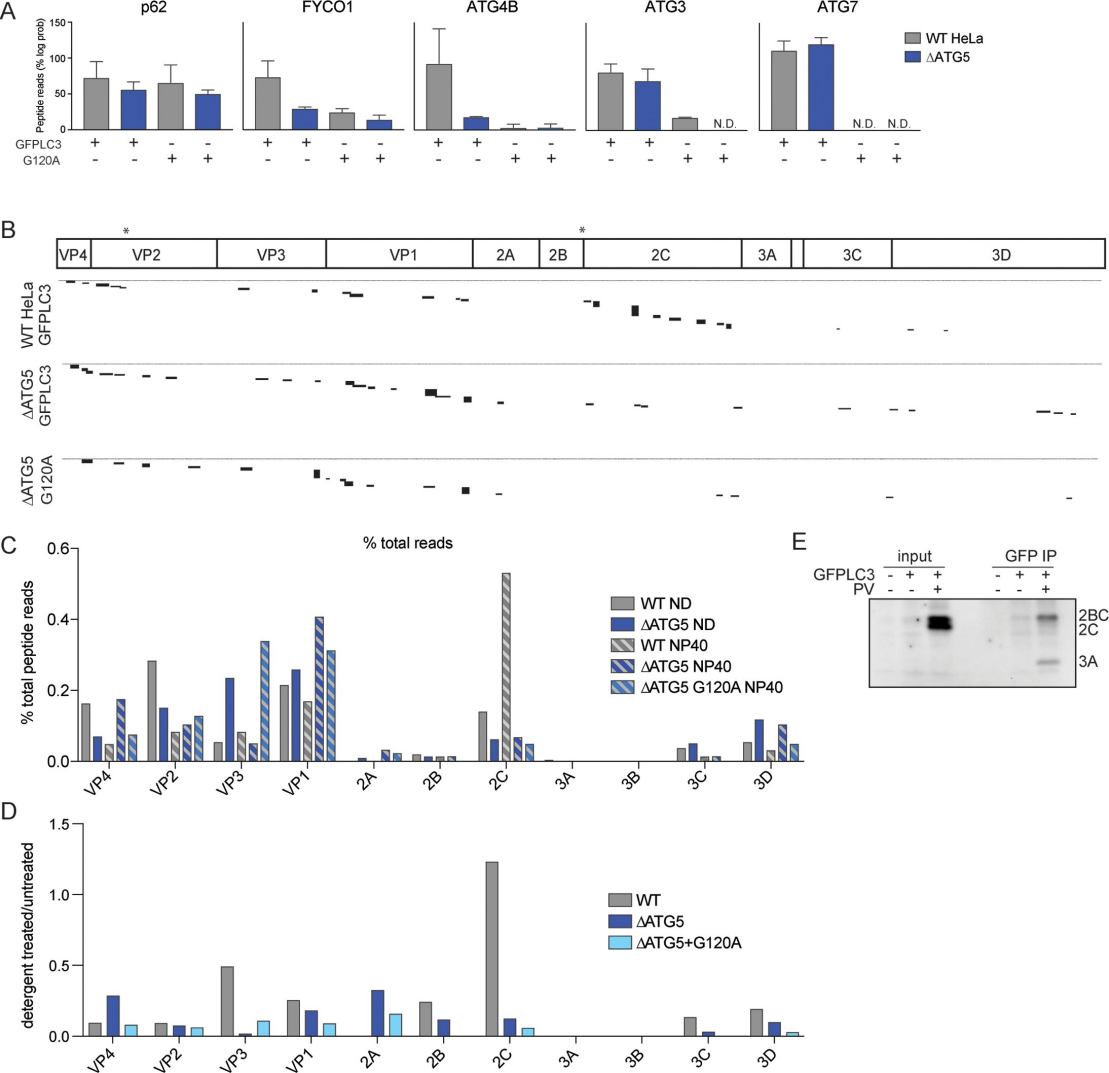
Viral proteins bind LC3. (A) Cells were transfected with GFP–LC3 or GFP–LC3–G120A for 48 hours and infected with PV at an MOI of 10 PFU/cell for 6 hours. An immunoprecipitation was done with anti-GFP antibodies, submitted for mass spectrometry, and peptide read abundance analyzed. Known LC3 interactors were assessed for binding capacity by comparing the log probability of peptide reads to uninfected control samples, which were set to 100%. N.D. indicates no peptide reads were detected. (B) Peptide reads from PV proteins were aligned to the PV genome. Black blocks indicate length and abundance of peptide reads. Stars indicate location of WxxL motifs in the PV genome. (C) PV peptide reads were analyzed by percent total reads for each of the viral proteins. (D) PV peptide reads were analyzed as total reads of detergent-treated samples over nontreated samples. (E) Cells were transfected with GFP–LC3 for 48 hours and infected with PV at an MOI of 10 PFU/cell for 6 hours. Cells were lysed with NP-40 buffer and immunoprecipitated with anti-GFP antibodies. Immunoblots were stained with antibodies against PV proteins 2C and 3A. See also S4 and S5 Figs and S6 Data. GFP, green fluorescent protein; LC3, light-chain 3; MOI, multiplicity of infection; PFU, plaque-forming units; PV, poliovirus.

To determine how PV leads to recruitment of unlipidated LC3 to membranes, we used an anti-GFP antibody to capture GFP–LC3 and tightly associated, detergent-resistant proteins during infection. Mass spectrometry (MS) was then used to identify the co-immunoprecipitated (IP) proteins. To confirm that our GFP–LC3 IP was specific, we compared several cellular proteins known to interact with both LC3-I and LC3-II or only with LC3-II (Fig 6A). The LC3 lipidation process starts with a C-terminal cleavage by the proteinase ATG4B and covalent attachment first to ATG7 and then to ATG3 [71–73]. The ATG5/12/16L complex then acts as an E3 ligase, transferring the covalent attachment of LC3 from ATG3 to PE [74]. LC3 binds to p62 whether it is lipidated or not [75,76], and, indeed, we observe equal binding to p62 in WT and Δ*ATG5* cells and with GFP–LC3 or GFP–LC3–G120. However, autophagy-associated protein FYCO1 only binds to LC3-II [77], as there were fewer peptide reads in Δ*ATG5* cells or when GFP–LC3–G120A was used. We saw little to no binding of ATG4B, which binds the terminal glycine of LC3 [72,73], when GFP–LC3–G120A was used. Interesting, ATG4B–LC3 binding was significantly reduced in Δ*ATG5* cells, perhaps because LC3-II is also one of its substrates [20,78]. ATG7 and ATG3, which participate in the covalent conjugation of LC3 upstream of the function of ATG5, bound to GFP–LC3 in the presence or absence of ATG5. However, the G120A substitution cannot initiate the cascade of covalent attachments and is not recognized by either ATG7 or ATG3 [79]. Thus, the recovery of peptides from all five of these LC3-interacting proteins exhibits differential dependence on the lipidation status of LC3 in accordance with their biological roles.

To identify viral proteins that directly bind to LC3 as well as those that are found on the same membranous surface, we prepared samples that did or did not contain detergent to disrupt membranes. Plasmids that expressed either GFP–LC3 or GFP–LC3–G120A were transfected into WT or Δ*ATG5* cells and infected with PV. The viral peptides identified by IP-MS were mapped across the viral genome to visualize the viral proteins pulled down with LC3 (Fig 6B). In the absence of detergent, peptides from almost every viral protein were recovered (S4A Fig). However, in the presence of detergent, most peptides pulled down from WT cells were derived from capsid proteins and 2C (Fig 6B). When we analyzed PV peptide reads as percentage of total reads, capsid proteins formed a large percentage of the total peptide reads in all conditions, suggesting that these viral proteins bind LC3 or LC3-associated membranes (Fig 6C). The ratio of reads in the presence of detergent to those in its absence showed that only peptides in 2C in WT cells remained associated with LC3 in the presence of detergent (Fig 6D). We hypothesize that LC3 binding to viral protein 2C, a known hydrophobic protein, is stabilized by LC3 lipidation.

To determine which form of protein 2C immunoprecipitated with GFP–LC3, we visualized the collected proteins by SDS PAGE and immunoblotted with anti-2C antibody (Fig 6E). Interestingly, precursor 2BC but not 2C was pulled down, arguing that the peptides identified by MS actually derived from 2BC. We additionally found 3A pulled down with LC3, an interesting observation because 2BC and 3A together can induce membrane rearrangements similar to those induced by viral infection [35], and lead to LC3-II accumulation outside of viral infection [80]. We did not find 2B or 3A peptides pulled down in our MS data, perhaps because these are small proteins and thus difficult to detect by MS.

A consensus LC3-interacting region (LIR) has been identified with a common WxxL motif, and contacts between this motif and LC3-binding protein p62 have been visualized by X-ray crystallography [81,82]. Only two potential LIR motifs were identified in the PV genome: WWKL in viral capsid VP2 and WQWL in viroporin 2B, marked in the genome schematic (Fig 6B). Their high conservation in picornaviruses is shown in S3B Fig. The existence of these sites is consistent with the specific recruitment of LC3 by viral proteins. The destruction of cellular protein p62 during PV infection was observed in the presence or absence of canonical autophagy, which normally accomplishes its degradation (S4C Fig), perhaps ensuring LC3 availability for viral protein binding during infection.

GFP–LC3 IP-MS was also performed on DENV-infected bovine hamster kidney (BHK) cells with WT GFP–LC3 and GFP–LC3–G120A. To confirm that the MS data were specific to LC3 lipidation conditions, we saw both GFP–LC3 and GFP–LC3–G120A bound to p62 but not to ATG3 or ATG7 when LC3–G120A was used (S5A Fig). All DENV proteins were pulled down with WT GFP–LC3 and GFP–LC3–G120A (S5B Fig). This is not surprising, given that DENV proteins are known to directly interact with each other [67,83,84], and suggests that one or more DENV proteins likely bind to LC3. Possible LIR motifs found in NS1, NS2A, and NS5 could potentially be sites of interaction with LC3.

## Discussion

**Fig 7:**
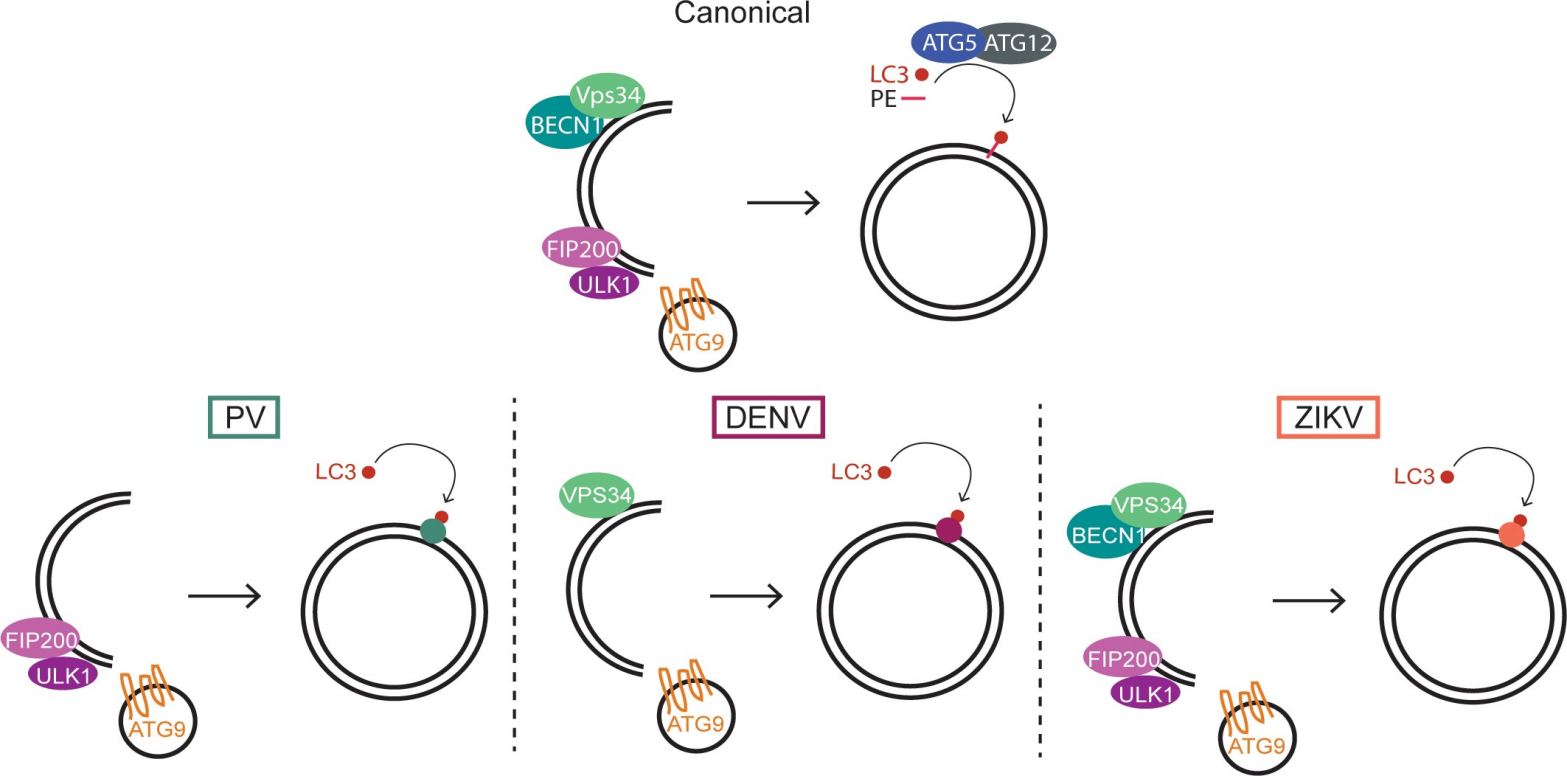
Model for viral utilization of autophagy pathway components. Canonical autophagy uses upstream components ULK1/FIP200 complex, VPS34/BECN1 complex, as well as ATG5-mediated lipidation of LC3. PV, DENV, and ZIKV use unique sets of components to induce different membrane structures for viral amplification and spread. ATG5, autophagy-related gene 5; BECN1, beclin-1; DENV, dengue virus; FIP200, PTK2/FAK family interacting protein of 200 kDa; LC3, light-chain 3; PV, poliovirus; ULK1, Unc-like autophagy-activating kinase; ZIKV, Zika virus.

By comparing how different RNA viruses manipulate autophagy factors, we show here that there are numerous ways by which a virus can hijack this complex cellular pathway. Each virus, even closely related viruses like DENV and ZIKV, uses slightly different components, although they might ultimately use the subsequent membranes and degradative processes for similar purposes. Each virus requires a different subset of initiation components for efficient viral growth, although all three viruses utilize lipid scavenger ATG9 and recruit LC3 directly to membranes, bypassing the need for ATG5-mediated lipidation (Fig 7). We believe that this comparative study addresses some of the inconsistencies seen with viral induction of the autophagy pathway.

The fact that the ULK1/FIP200 and BECN1/VPS34 complexes can be bypassed by individual viruses is consistent with the idea that noncanonical autophagy pathways can be induced in response to different stress stimuli [85]. ULK1-independent autophagy can be induced by glucose deprivation or excessive ammonia [23,86]. BECN1-independent autophagy can be induced by proapoptotic compounds such as Z18 [24,87]. There is also evidence for a noncanonical autophagy pathway that is VPS34-dependent and BECN1-independent upon induction by arsenic trioxide [88], providing precedent for the DENV-induced noncanonical pathway. These data highlight that functional cellular autophagy is not entirely reliant on every component of the canonical pathway. Perhaps many viruses take advantage of these alternate and possibly redundant pathways to ensure a productive infection [89].

One of the striking findings described here is the dependence of PV, DENV, and ZIKV infection on LC3 but not on its lipidation. It has been shown previously that unlipidated LC3-I is found on viral-associated vesicles during infection with murine hepatitis virus, equine arteritis virus, and Japanese encephalitis virus [90–92]. These data, in addition to LC3-dependent and ATG7-independent viral amplification, led these authors to conclude that the virally induced membranes were not derived from the autophagy pathway. Instead, colocalization of ER degradation enhanced by alpha-mannosidase (EDEM1) and LC3 suggested that the virally induced vesicles were ER-associated protein degradation (ERAD) associated [90]. We argue that direct recruitment of LC3 to membranes by viruses can perform autophagosome-like functions. Colocalization with EDEM1 could be a result of the known degradation of EDEM1-containing vesicles by an autophagy-like process [93]. We specifically found unlipidated LC3 associated with membranes during viral infection as well as direct binding of LC3 to PV proteins by IP-MS. A few cases of cellular LC3 lipidation–independent autophagy have been described [25,94]. In these cases, when either ATG5 or ATG7 is ablated, the autophagy pathway can still proceed, leading to the formation of autophagosomes in response to unusual stimuli, such as the drug etoposide [25]. LC3 is not lipidated under these conditions. It remains unknown how these autophagosomes fuse without lipidated LC3. LC3-I has been found associated with membranes during cellular secretory processes, although how LC3-I is recruited to membranes was not tested [93]. It is possible that in these lipidation-independent cases protein-mediated recruitment of LC3-I occurs, as we observed during viral infection.

Recently, the influenza M2 protein was found to contain a functional LIR motif. This LIR motif was shown to be important for viral-mediated relocalization of LC3 to the plasma membrane, presumably to facilitate viral budding [95]. This represents a striking example of autophagosome relocalization by a virus. We have identified potentially functional viral LIR motifs in the capsid protein VP2 and 2BC protein of PV, as well as in the polyproteins of DENV and ZIKV. One hypothesis is that PV proteins bind LC3 as a means to localize PV-replication complexes and capsids specifically to autophagosome-like membranes for assembly purposes. 2BC is localized to replication complexes and is a membrane-associated protein [96], so an interaction with LC3 at the outer surface of the double membrane is possible. The binding of capsid proteins to LC3 was unanticipated but of potential functional interest. Immature capsid precursors are known to be specifically associated with membranes [97] and intact virions can be released nonlytically by a process similar to secretory autophagy [39,40,98]. We propose that LC3 association of 2BC and immature capsids facilitate RNA replication, RNA packaging, and intercellular spread.

Autophagosome-dependent unconventional secretion was first demonstrated in yeast [99–101]. In human cells, autophagy-dependent secretion has been demonstrated for interleukin 1β [102] and increasing numbers of other proteins and complexes [26,103]. This suggests that autophagosomes can be rerouted to the plasma membrane and the contents released, providing precedent for virally induced double-membraned structures exiting a cell nonlytically. Infectious picornaviruses such as PV, Coxsackievirus B, and Hepatitis A virus have been found to be released in membrane-bound vesicles, each containing many viral particles [40,41,104]. For both unconventional secretion and the assembly of intracellular membranous components, the cellular autophagy pathway, with its unusual topologies and induction of membrane curvatures, is a rich source of host components for many viral infections.

We observed in mice that fasting could enhance viral growth of PV. We also show that the increase in PV titers is dependent upon the presence of Atg5, linking the starvation phenotype to its role in inducing the autophagy pathway. The data presented here show that PV benefits from the induction of the canonical autophagy pathway but do not require all of the components when virally inducing the pathway. We hypothesize that pre-existing membrane structures from starvation-induced autophagy can be utilized by viruses for growth and maturation, leading to quicker exit from cells and overall more virus production. Malnutrition has been linked to increased death from infectious diseases in children [105], and, although exacerbation of disease by malnutrition is often attributed to confounding effects, another outcome of malnutrition is induction of cellular autophagy. The implications of these data are broad: if viral infection is exacerbated by induction of the autophagy pathway, then starvation and many over-the-counter drugs that induce autophagy could lead to greater disease severity. It also implies that simple supplementation of nutrients to block induction of the autophagy pathway may help abrogate increased viral amplification.

## Materials and methods

### Cells and viruses

Human HeLa cells and BHK21 cells were cultured in Dulbucco’s modified eagle’s medium (Gibco) supplemented with 10% bovine serum and 1% penicillin/streptomycin. PV type 1 Mahoney was grown from infectious cDNA, as previously described [106]. DENV type 2 (16681) was propagated from an infectious cDNA clone (pD2/IC) in C6/36 mosquito cells. The ZIKV strain used was PRVABC59 (Puerto Rico). The virus was purchased from BEI Resources and propagated on C6/36 mosquito cells.

### CRISPR-Cas9 KO cell line generation

CRISPR-Cas9 plasmid px458 (Addgene #48138) was cut with BbsI and ligated to annealed sgRNAs (see S1 Table), as described previously [107]. HeLa cells were transfected with resulting plasmids (Lipofectamine 3000, Invitrogen). Forty-eight hours later, cells were single-cell sorted for GFP+ cells using a BD Aria II sorter (Stanford FACS facility) in a 96-well format. Cells that propagated were tested for gene disruption by harvesting genomic DNA, PCR amplifying the region of interest, and sequencing to look for the presence of frameshift mutations. Clones were also tested for reduced protein expression by western blot. For the *ATG9*, *BECN1*, *VPS34*, and *LC3B* genes, multiple KO lines were obtained and tested, although subsequent experiments were carried out using one representative line.

### Confocal microscopy

Cells were transfected with 50 ng/well of GFP–LC3 in a 24-well plate. After 24 hours, cells were seeded on coverslips and infected or treated the following day. Cells were fixed in 4% paraformaldehyde, stained with DAPI (Invitrogen), and mounted on slides with PermaFluor (Thermo Fisher Scientific). Slides were imaged using a Leica SP8 confocal microscope (Stanford imaging facility). GFP–LC3 puncta were counted per cell.

### Flow cytometry and viral-specific probes

Infected cells from a 24-well plate were fixed and stained using the PrimeFlow RNA assay according to the manufacturer’s protocol (Invitrogen). Viral RNA-specific probes were used for the positive strand of PV (VF1-10252), DENV2 (VF1-15158), or ZIKV (VF1-20236). Cells were run on a flow cytometer (Scanford, Stanford FACS facility) and analyzed using FlowJo software (v. 10.4).

### RT-qPCR

Total RNA was isolated from cells using the RNeasy mini kit (Qiagen). RT-qPCR was performed on an Applied Biosystems 7300 machine using the QuantiTect SYBR Green RT-qPCR Kit (Qiagen). Primers specific to PV, DENV, or ZIKV were used (see S2 Table).

### Luciferase assay

Cells were plated in a 96-well plate and transfected with 8 ng of PV replicon RNA (kind gift from Raul Andino). A subset of cells were treated with 2 mM guanidine (Sigma) prior to transfection to inhibit viral replication. Cell lysates were collected at 0, 2, 4, and 6 hours post transfection, and firefly luciferase activity was measured using the Firefly Luciferase Assay System (1000) in a 96-well format with a BioTek Neo2 luciferase reader.

### Immunoblotting

Protein lysates were harvested with RSB buffer (10 mM NaCl, 10 mM Tris pH 7.5, 1.5 mM MgCl_2_, and 1% NP-40) with added EDTA-free protease inhibitor (Roche). Lysates were quantified using a DC protein assay (Bio-Rad), run on an SDS PAGE gel, and transferred to PVDV membranes. Immunoblots were blocked in 5% milk or BSA for phospho antibodies and incubated overnight with anti-LC3 antibody (Novus biologicals); PV 2C and 3A antibodies (kindly provided by Ellie Erhenfeld and Kurt Bienz); DENV NS3 antibody (GeneTex); p62 antibody (Sigma); FIP200 antibody (Abcam); ULK1, BECN1, VPS34, ATG9, and ATG5 antibodies (Cell Signaling); GAPDH antibody (Santa Cruz); phospho-AMPK alpha-1 (Thermo Fisher); and phospho-p70 S6K (Cell Signaling) at 1:1,000 dilution. Secondary antibodies conjugated to HRP (Invitrogen) were used at 1:10,000 dilution. Immunoblots were imaged on a ChemiDoc (Bio-Rad).

### Tranfections and Lentiviral transductions

DNA transfections were done using a Lipofectamine 3000 kit (Invitrogen). The ULK1 plasmid was acquired from Addgene (#31963). The K46I mutation was made by site-directed mutagenesis (QuickChange Lightening kit, Agilent). Lentiviral vectors were cloned using Gibson cloning (NEB), inserting the gene of interest into the pLenti-CMV-Puro-DEST vector, as described previously [108]. 293T cells were transfected with the pLenti vector containing the addback gene, Gag-Pol, VSV-G, and p-Advent. Supernatants were collected 24 hours later and treated with protamine sulphate. Supernatants were added to KO cell lines and allowed to infect for 24–48 hours. Successful transductions were selected by 2 μg/ml puromycin for up to 1 week. Resulting cell lines were tested for rescue of protein levels by western blot. For siRNA knock-down of ULK1 and ULK2, 4 pooled siRNAs targeting human ULK1 or ULK2 (siGENOME SMARTpool, Dharmacon #005049 and #005396) were transfected into HeLa cells using Lipofectamine 3000 (Life Technologies). Knockdown was tested by qPCR at 72 hours post transfection.

### Electron microscopy

WT and KO cell lines were plated in 10-cm plates and infected with PV (6 hours) or DENV (24 hours) at an MOI of 10 PFU/cell. A subset of samples were infected and treated with 2 mM guanidine (Sigma) simultaneously. Cells were collected and fixed with 4% PFA for 10 minutes. Cells were washed twice with DMEM and resuspended in 50 ul of 20% BSA in DMEM. Samples were subjected to high-pressure freezing using a Leica EmPACT High Pressure Freezer and freeze substitution to replace water with acetone (Stanford Imaging Facility). Samples were sectioned and imaged on a transmission electron microscope (JEOL JEM1400, Stanford Imaging Facility). Per condition, 10–20 cells were analyzed for cellular structures on blinded images.

### Autophagy inducers and inhibitors

ULK1 inhibitor MBL56 (kind gift from Kevin Shokat) was used at 0.5, 5, and 50 μM. Rapamycin was used at 1 μM in DMSO (Sigma); Chloroquine (CQ) was used at 50 μM (Sigma); Spautin-1 was used at 10 μM (Sigma); SAR405 was used at 1, 5, and 10 μM (Sigma); and Hanks’ Balanced Salt Solution (HBSS; Gibco) plus 10% FBS was used for starvation media.

### Immunoprecipitations and mass spectrometry

GFP immunoprecipitations were performed with GFP-Trap magnetic beads (Chromotek). Cells were harvested with lysis buffer (10 mM Tris pH 7.5, 150 mM NaCl, 0.5 mM EDTA, and 0.5% NP-40) containing EDTA-free protease inhibitor (Roche). Magnetic beads were added and allowed to bind for 2 hours at 4°C with rotation. Beads were washed 3 times with wash buffer (10mM Tris pH 7.5, 150 mM NaCl, and 0.5 mM EDTA) and eluted with 2 M glycine pH 2.5, followed by neutralization with 1 M Tris pH 10.4, for mass spectrometry. Samples analyzed by immunoblot were eluted by boiling in 2X SDS sample buffer (120 mM Tris pH 6.8, 20% glycerol, 4% SDS, 0.04% bromophenol blue, and 10% BME) and run on an SDS PAGE gel. Mass spectrometry was performed and analyzed by the Stanford University Mass Spectrometry facility (SUMS).

### Mouse infections

Embryos of conditional Atg5 flox/+ mice B6.129S (Hara et al., 2006) were obtained from Dr. Noboru Mizushima through the RIKEN BioResource Center (Ibaraki, Japan; stock RBRC02975) and cryorecovered at Stanford University. Atg5 flox/+ mice were further backcrossed to C57BL/6J to N10. B6.Cg-Ndor1Tg(UBC-cre/ERT2)1Ejb/J, a lentitransgenic line harboring a single-copy integrant of a fusion gene consisting of Cre recombinase and a mutant human estrogen receptor (ERT2) under control of the human ubiquitin C promotor (UBC), was obtained from the Jackson Laboratory (Bar Harbor, Maine; stock 008085) at N6 and further backcrossed to C57BL/6J to N10.

C57BL/6 mice expressing the human PVR, *Atg5*^fl/fl^, and Cre^+/-^ were treated with 75 mg/kg of tamoxifen for 5 days, followed by 2–4 weeks of recovery to induce the expression of Cre and subsequent excision of the floxed *Atg5* exon 2. Ten to twelve-week-old mice were fasted for 48 hours prior to infection or given 25 mg/kg loperamide (Sigma) in saline by i.p. injection every 12 hours for the duration of the infection. Mice were infected intramuscularly with PV (1 x 10^7^ PFU/50 ul in PBS). Muscle tissue was harvested 4 dpi and processed using the Bullet Blender BBX24 (Next Advance). DNA and protein lysate were also extracted from tissues, using the DNeasy Blood and Tissue kit (Qiagen) and RSB buffer, respectively.

### Ethics statement

The mouse experiments in this study were approved by the Administrative Panel on Laboratory Animal Care at Stanford University (approval number 9296). This committee is accredited by the Association for the Assessment and Accreditation of Laboratory Animal Care (AAALAC International).

## Supporting information

S1 Fig Related to Fig 1. Characterization of autophagy KO cells. (A) Sequence analysis of CRISPR-Cas9 KO cell lines. Genomic DNA was harvested from HeLa cells and the gene of interest PCR amplified around the targeted cut site. PCR samples were sequenced by Sanger sequencing. The guide RNA and PAM recognition sequences are indicated, and arrows above the chromatogram traces show where in the sequence the KO cells was altered. Additional TOPO cloning shows the exact deletion for each allele. Numbers indicate frequency of alleles among tested colonies. (B) Protein lysates were run on SDS PAGE gels and immunoblotted for the proteins of interest. GAPDH was used as a loading control. (C) Cells were starved in HBSS media for 2 hours or left untreated. Cells were fixed and stained for endogenous LC3 with an anti-LC3 antibody, followed by a secondary antibody conjugated to Alexa488. Samples were visualized by confocal microscopy, and puncta per cell were quantified; *n* = 40 cells. Representative images are shown from WT and one KO cell line. All data are represented as mean +/− SEM. *Indicates significant *P* value of <0.05, **P value < 0.01, ***P value < 0.001, ****P value > 0.0001 by a Mann–Whitney test. CRISPR, Clustered Regularly Interspaced Short Palindromic Repeats; GAPDH, glyceraldehyde 3-phosphate dehydrogenase; HeLa, human epithelial-derived cell line; KO, knockout; LC3, light-chain 3; PAM, protospacer adjacent motif; WT, wild-type.
(TIF)

S2 Fig Related to Fig 1. Viral infections of autophagy KO cells and mice. (A) WT or *ΔULK1* cells were transfected with an empty vector or a plasmid containing ULK1 or ULK1–K46I for 48 hours. *ΔFIP200* cells were transduced with a pLentiviral vector expressing FIP200. Cells were infected with PV at an MOI of 0.1 PFU/cell and harvested at 6 hpi. (B) siRNAs against ULK2 were transfected into WT or *ΔULK1*, or siRNAs against ULK1 and ULK2 were transfected into *ΔFIP200* cells. RT-qPCR was performed on RNA. Cells were infected with DENV at an MOI of 0.1 PFU/cell and supernatant titered at 24 hpi. (C) *ΔVPS34* cells were transduced with a pLentiviral vector expressing VPS34. Cells were infected with DENV at an MOI of 0.1 PFU/cell for 24 hours. (D) C57BL/6 mice expressing PVR^+/+^ ATG5^fl/fl^Cre^−/−^ or PVR^+/+^ ATG5^fl/fl^Cre^+/−^ were treated with tamoxifen and infected intramuscularly with PV for 4 days. Calf muscle tissue was harvested, and DNA was extracted. qPCR was done for the indicated regions of the Atg5 gene. (E) The same mouse tissue as above was also used to extract protein lysates, run on an SDS PAGE gel and blotted for LC3. All data are represented as mean +/− SEM. *Indicates significant *P* value of <0.05, **P value < 0.01, ***P value < 0.001, ****P value > 0.0001 by an unpaired *t* test. ATG5, autophagy-related gene 5; DENV, dengue virus; F, female mice; FIP200, PTK2/FAK family interacting protein of 200 kDa; hpi, hours post infection; K46I, kinase dead ULK1 mutant; KO, knockout; LC3, light-chain 3; M, male mice; MOI, multiplicity of infection; PFU, plaque-forming units; PV, poliovirus; PVR, poliovirus receptor; RT-qPCR, reverse transcription quantitative PCR; siRNA, small interfering RNA; ULK, Unc-like autophagy-activating kinase; WT, wild-type.
(TIF)

S3 Fig Related to Fig 2. Viral entry and protein abundance. (A) Cells were infected with PV at an MOI of 0.1 PFU/cell for 30 minutes, then washed with citric acid wash and PBS 3 times. RNA was harvested, and RT-qPCR was done for viral RNA, normalized to GAPDH. (B) HeLa cells were infected with PV at MOI 0.1 PFU/cell and protein lysates harvested at the indicated times. Lysates were run on an SDS PAGE gel and immunoblotted for PV 2C and GAPDH. (C and D) Cells were transfected with PV replicon and harvested at the times indicated. Luciferase expression was analyzed as Firefly RLU. (E) Cells were infected with DENV at MOI 0.1 PFU/cell, protein lysates harvested, and immunoblotted for DENV NS3 and GAPDH. DENV, dengue virus; GAPDH, glyceraldehyde 3-phosphate dehydrogenase; HeLa, human epithelial-derived cell line; MOI, multiplicity of infection; PFU, plaque-forming unit; PV, poliovirus; RLU, relative luciferase units; RT-qPCR, reverse transcription quantitative PCR.
(TIF)

S4 Fig Related to Fig 6. PV proteins bind LC3, and LIR domains are conserved. (A) Cells were transfected with GFP–LC3 for 48 hours and infected with PV at an MOI of 10 for 6 hours. Cells were mechanically lysed by douncing in buffer without NP-40. A GFP IP was performed, and the eluent was sent for MS. Peptide reads from viral proteins were aligned to the viral genome. (B) Viral protein VP2 and 2B alignments done by Clustal Omega for four picornaviruses: PV, RHV-1a, CVB3, and EV70. Red boxes indicate the WxxL LIR motifs. (*) indicates full conservation, (:) indicates partial conservation with similar amino acids, and (.) indicates partial conservation. (C) WT cells were treated with Rap (6 hours), Spautin-1 (24 hours), or CQ (4 hours) and infected with PV (MOI 1.0 PFU/cell) for 6 hours. Cell lysates were run on SDS PAGE and blotted for p62 and GAPDH. CVB3, Coxsackievirus B3; CQ, chloroquine; EV70, Enterovirus 70; GAPDH, glyceraldehyde 3-phosphate dehydrogenase; GFP, green fluorescent protein; IP, immunoprecipitated; LC3, light-chain 3; LIR, LC3-interacting region; MOI, multiplicity of infection; MS, mass spectrometry; PFU, plaque-forming units; PV, poliovirus; Rap, rapamycin; RHV-1a, Rhinovirus 1a; WT, wild-type.
(TIF)

S5 Fig Related to Fig 6. DENV proteins bind LC3. (A) BHK21 cells were transfected with GFP–LC3 or GFP–LC3–G120A for 48 hours and infected with DENV (MOI 10 PFU/cell) for 24 hours. Cells were lysed with buffer containing NP-40 and a GFP IP performed. Eluent was submitted for mass spectrometry and binding capacity assessed by comparing the log probability of peptide reads from infected samples to uninfected control samples, which were set to 100%. N.D. indicates no peptide reads were detected. (B) DENV peptide reads from the GFP–LC3 IP-MS were aligned to the DENV genome from LC3 and LC3–G120A samples. Stars indicate the location of WxxL LIR motifs in the DENV genome. BHK, bovine hamster kidney; DENV, dengue virus; GFP, green fluorescent protein; IP, immunoprecipitated; LC3, light-chain 3; LIR, LC3-interacting region; MOI, multiplicity of infection; MS, mass spectrometry; PFU, plaque-forming units.
(TIF)

S1 Table Related to Fig 1 and S1 Fig. Sequences for guide RNAs. Nucleotides in bold denote the added cut site for the BBS1 restriction endonuclease site.
(DOCX)

S2 Table Related to Fig 2 and S1 Fig. Primer sequences for quantitative PCR. Primers used for viral genomic RNA and for the murine ATG5 gene that contained the flox sites required for CRE-specific clevage.
(DOCX)

S1 Data (XLSX)

S2 Data (XLSX)

S3 Data (XLSX)

S4 Data (XLSX)

S5 Data (XLSX)

S6 Data (XLSX)

## Abbreviations

3-MA: 3-methyladenine
AMPK: AMP-activated protein kinase
ATG: autophagy-related gene
BECN1: beclin-1
BHK: bovine hamster kidney
CRISPR: Clustered Regularly Interspaced Short Palindromic Repeats
CQ: chloroquine
CVB3: Coxsackievirus B3
DENV: dengue virus
DMV: double-membraned vesicle
EDEM1: ER degradation enhanced by alpha-mannosidase
EM: electron microscopy
ER: endoplasmic reticulum
ERAD: ER-associated protein degradation
FIP200: PTK2/FAK family interacting protein of 200 kDa
GAPDH: glyceraldehyde 3-phosphate dehydrogenase
GFP: green fluorescent protein
HeLa: human epithelial-derived cell line
hpi: hours post infection
IP: immunoprecipitated
K46I: kinase dead ULK1 mutant
KO: knockout
LC3: light-chain 3
LIR: LC3-interacting region
MOI: multiplicity of infection
MS: mass spectrometry
mTOR: mammalian target of rapamycin
MVB: multivesicular body
PAM: protospacer adjacent motif
PE: phosphatidylethanolamine
PFU: plaque-forming units
PV: poliovirus
PVR: poliovirus receptor
qPCR: quantitative PCR
Rap: rapamycin
RLU: relative luciferase units
RT-qPCR: reverse transcription quantitative PCR
siRNA: small interfering RNA
ULK: Unc-like autophagy-activating kinase
WT: wild-type
ZIKV: Zika virus
