# Supplementary figures and images for: Differential and convergent utilization of autophagy components by positive-strand RNA viruses
Source: PLoS Biol. 2019 Jan 4;17(1):e2006926. doi: 10.1371/journal.pbio.2006926 (PMC6334974; doi:10.1371/journal.pbio.2006926)

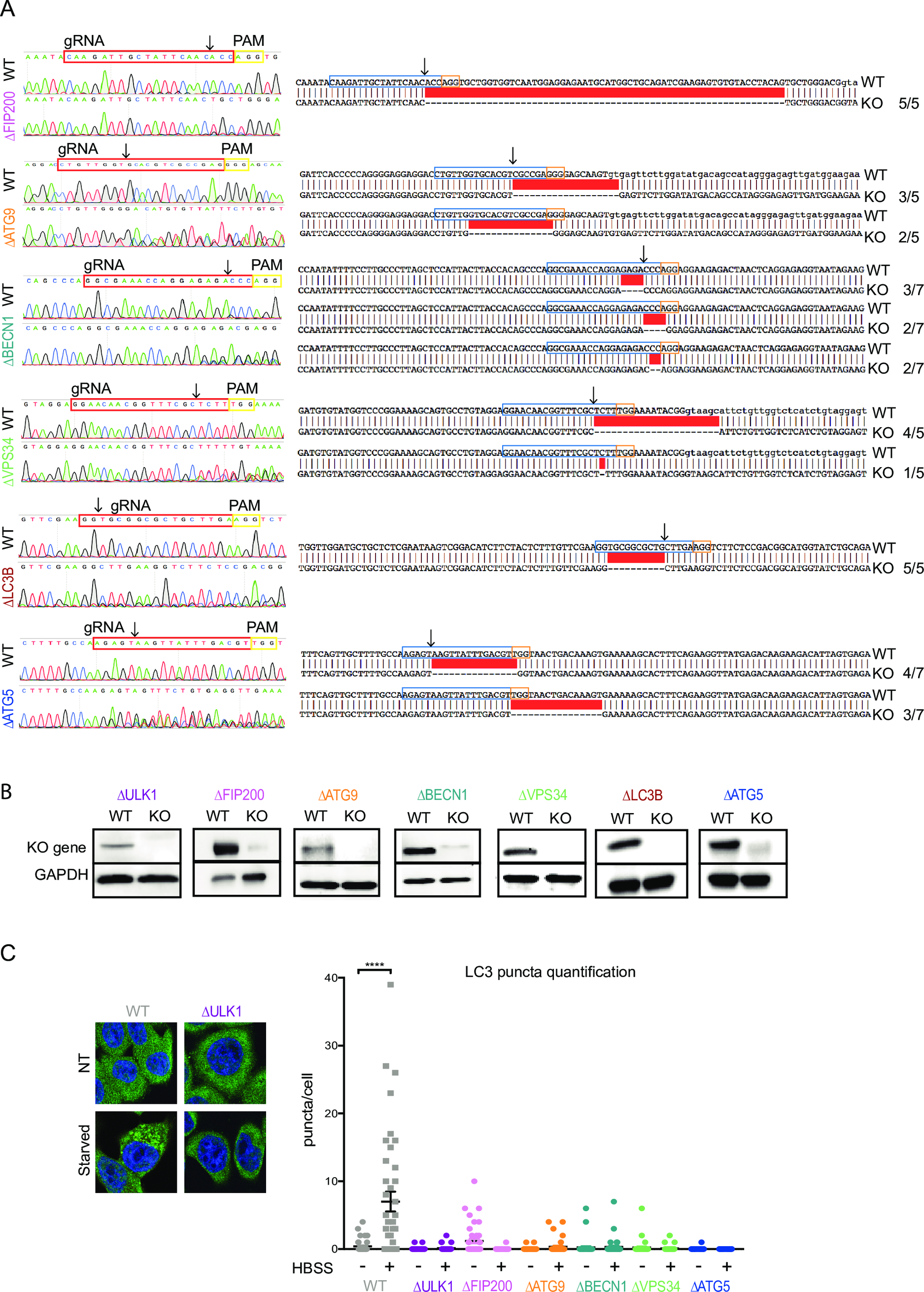

Supplement: S1 Fig — Characterization of autophagy KO cells. (A) Sequence analysis of CRISPR-Cas9 KO cell lines. Genomic DNA was harvested from HeLa cells and the gene of interest PCR amplified around the targeted cut site. PCR samples were sequenced by Sanger sequencing. The guide RNA and PAM recognition sequences are indicated, and arrows above the chromatogram traces show where in the sequence the KO cells was altered. Additional TOPO cloning shows the exact deletion for each allele. Numbers indicate frequency of alleles among tested colonies. (B) Protein lysates were run on SDS PAGE gels and immunoblotted for the proteins of interest. GAPDH was used as a loading control. (C) Cells were starved in HBSS media for 2 hours or left untreated. Cells were fixed and stained for endogenous LC3 with an anti-LC3 antibody, followed by a secondary antibody conjugated to Alexa488. Samples were visualized by confocal microscopy, and puncta per cell were quantified; n = 40 cells. Representative images are shown from WT and one KO cell line. All data are represented as mean +/− SEM. *Indicates significant P value of <0.05, **P value < 0.01, ***P value < 0.001, ****P value > 0.0001 by a Mann–Whitney test. CRISPR, Clustered Regularly Interspaced Short Palindromic Repeats; GAPDH, glyceraldehyde 3-phosphate dehydrogenase; HeLa, human epithelial-derived cell line; KO, knockout; LC3, light-chain 3; PAM, protospacer adjacent motif; WT, wild-type. (TIF) [file pbio.2006926.s001.tif]

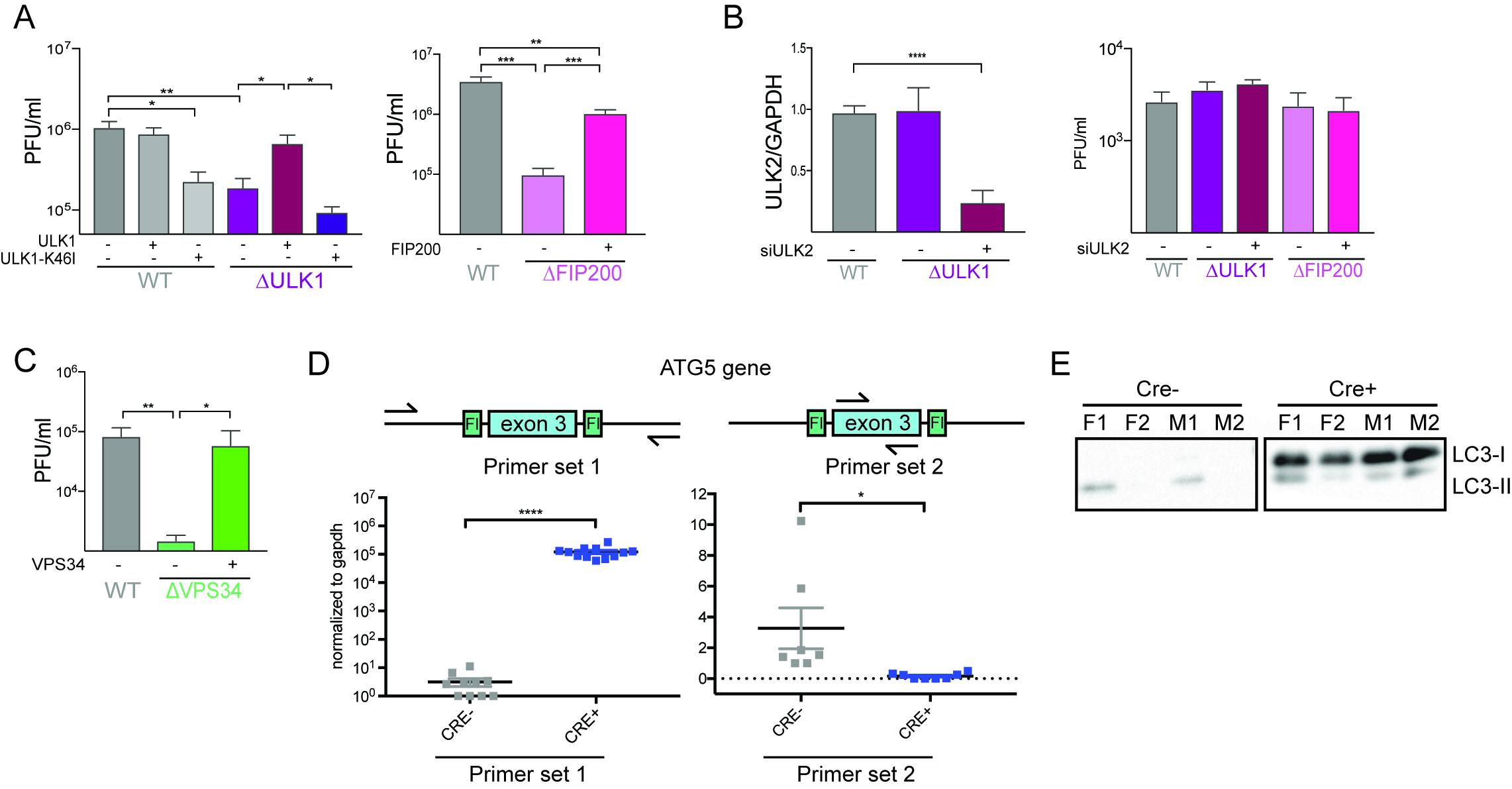

Supplement: S2 Fig — Viral infections of autophagy KO cells and mice. (A) WT or ΔULK1 cells were transfected with an empty vector or a plasmid containing ULK1 or ULK1–K46I for 48 hours. ΔFIP200 cells were transduced with a pLentiviral vector expressing FIP200. Cells were infected with PV at an MOI of 0.1 PFU/cell and harvested at 6 hpi. (B) siRNAs against ULK2 were transfected into WT or ΔULK1, or siRNAs against ULK1 and ULK2 were transfected into ΔFIP200 cells. RT-qPCR was performed on RNA. Cells were infected with DENV at an MOI of 0.1 PFU/cell and supernatant titered at 24 hpi. (C) ΔVPS34 cells were transduced with a pLentiviral vector expressing VPS34. Cells were infected with DENV at an MOI of 0.1 PFU/cell for 24 hours. (D) C57BL/6 mice expressing PVR+/+ ATG5fl/flCre−/− or PVR+/+ ATG5fl/flCre+/− were treated with tamoxifen and infected intramuscularly with PV for 4 days. Calf muscle tissue was harvested, and DNA was extracted. qPCR was done for the indicated regions of the Atg5 gene. (E) The same mouse tissue as above was also used to extract protein lysates, run on an SDS PAGE gel and blotted for LC3. All data are represented as mean +/− SEM. *Indicates significant P value of <0.05, **P value < 0.01, ***P value < 0.001, ****P value > 0.0001 by an unpaired t test. ATG5, autophagy-related gene 5; DENV, dengue virus; F, female mice; FIP200, PTK2/FAK family interacting protein of 200 kDa; hpi, hours post infection; K46I, kinase dead ULK1 mutant; KO, knockout; LC3, light-chain 3; M, male mice; MOI, multiplicity of infection; PFU, plaque-forming units; PV, poliovirus; PVR, poliovirus receptor; RT-qPCR, reverse transcription quantitative PCR; siRNA, small interfering RNA; ULK, Unc-like autophagy-activating kinase; WT, wild-type. (TIF) [file pbio.2006926.s002.tif]

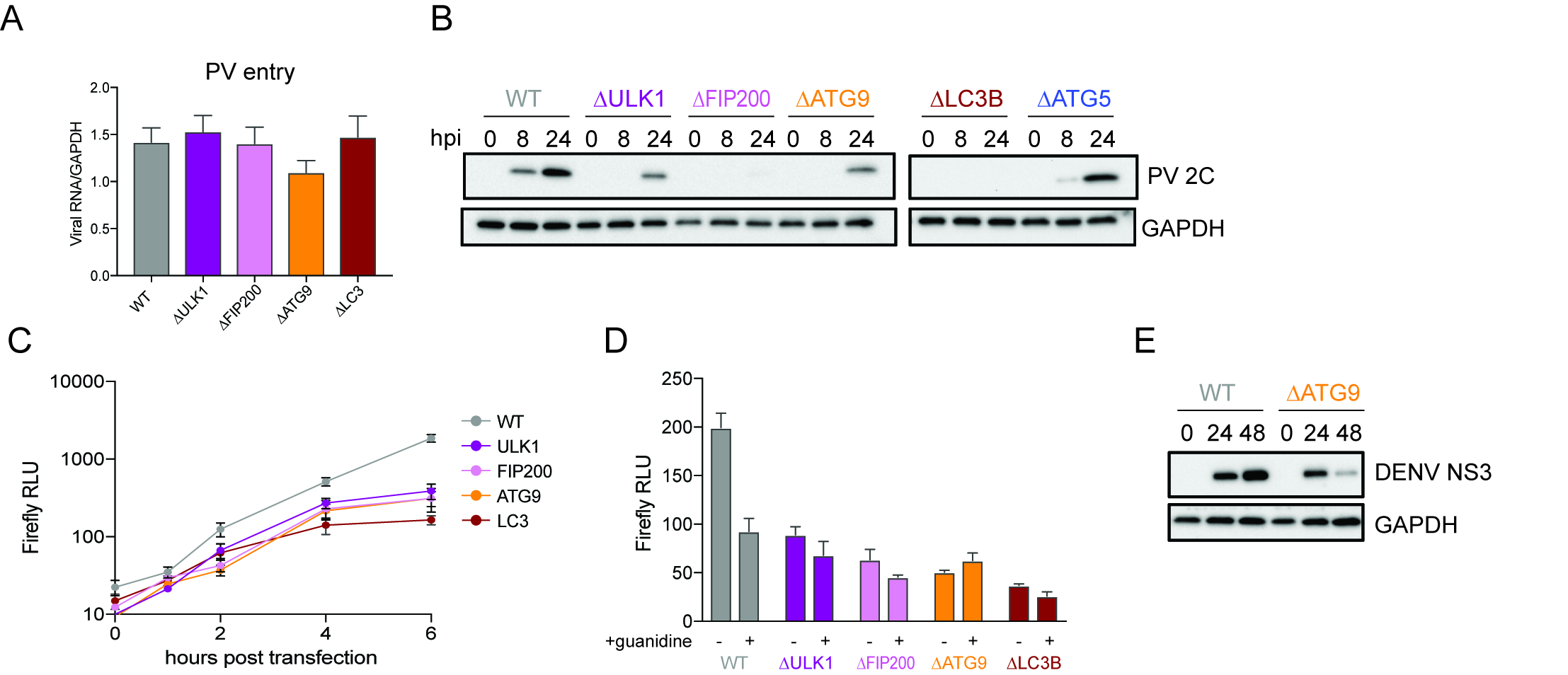

Supplement: S3 Fig — Viral entry and protein abundance. (A) Cells were infected with PV at an MOI of 0.1 PFU/cell for 30 minutes, then washed with citric acid wash and PBS 3 times. RNA was harvested, and RT-qPCR was done for viral RNA, normalized to GAPDH. (B) HeLa cells were infected with PV at MOI 0.1 PFU/cell and protein lysates harvested at the indicated times. Lysates were run on an SDS PAGE gel and immunoblotted for PV 2C and GAPDH. (C and D) Cells were transfected with PV replicon and harvested at the times indicated. Luciferase expression was analyzed as Firefly RLU. (E) Cells were infected with DENV at MOI 0.1 PFU/cell, protein lysates harvested, and immunoblotted for DENV NS3 and GAPDH. DENV, dengue virus; GAPDH, glyceraldehyde 3-phosphate dehydrogenase; HeLa, human epithelial-derived cell line; MOI, multiplicity of infection; PFU, plaque-forming unit; PV, poliovirus; RLU, relative luciferase units; RT-qPCR, reverse transcription quantitative PCR. (TIF) [file pbio.2006926.s003.tif]

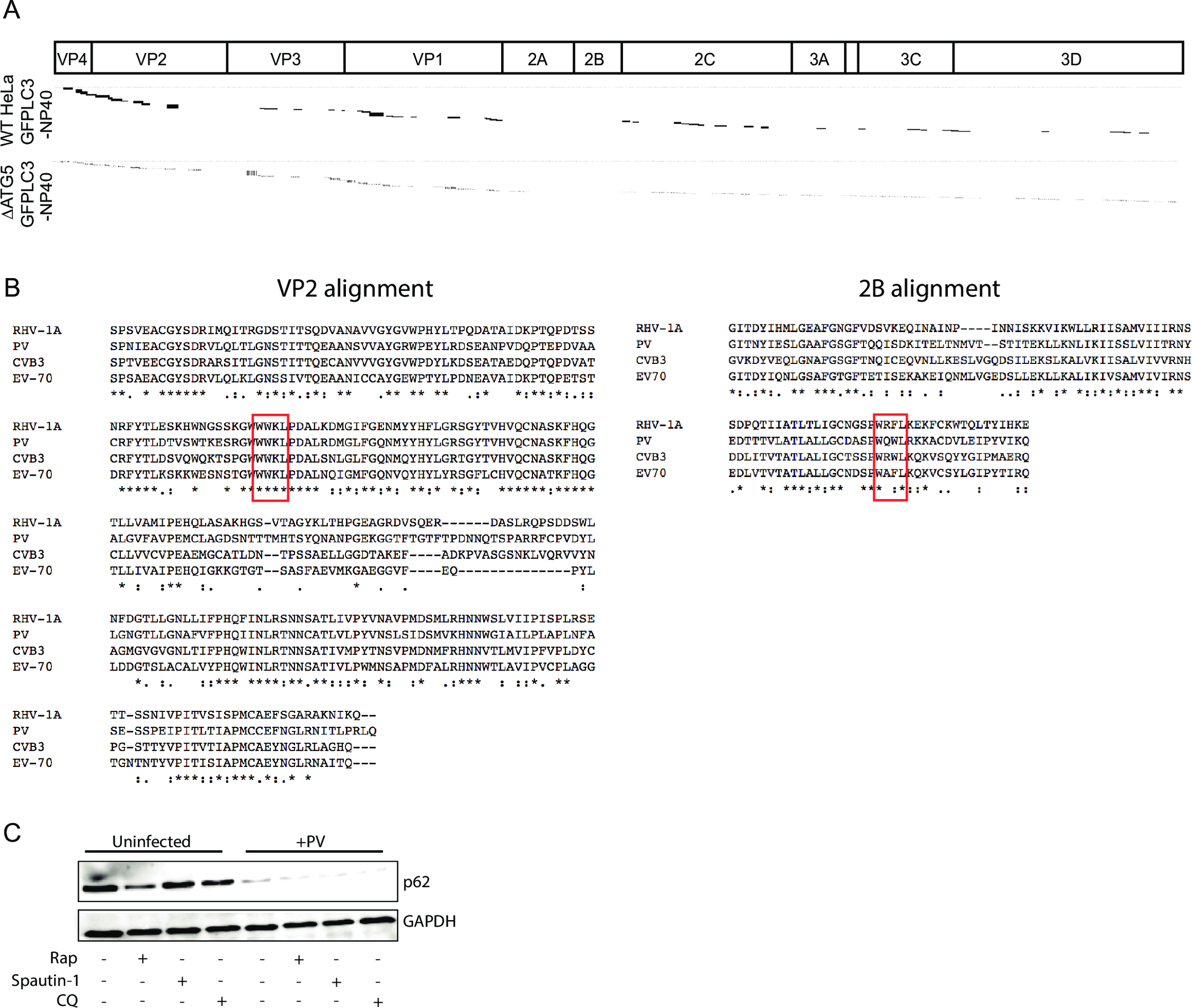

Supplement: S4 Fig — PV proteins bind LC3, and LIR domains are conserved. (A) Cells were transfected with GFP–LC3 for 48 hours and infected with PV at an MOI of 10 for 6 hours. Cells were mechanically lysed by douncing in buffer without NP-40. A GFP IP was performed, and the eluent was sent for MS. Peptide reads from viral proteins were aligned to the viral genome. (B) Viral protein VP2 and 2B alignments done by Clustal Omega for four picornaviruses: PV, RHV-1a, CVB3, and EV70. Red boxes indicate the WxxL LIR motifs. (*) indicates full conservation, (:) indicates partial conservation with similar amino acids, and (.) indicates partial conservation. (C) WT cells were treated with Rap (6 hours), Spautin-1 (24 hours), or CQ (4 hours) and infected with PV (MOI 1.0 PFU/cell) for 6 hours. Cell lysates were run on SDS PAGE and blotted for p62 and GAPDH. CVB3, Coxsackievirus B3; CQ, chloroquine; EV70, Enterovirus 70; GAPDH, glyceraldehyde 3-phosphate dehydrogenase; GFP, green fluorescent protein; IP, immunoprecipitated; LC3, light-chain 3; LIR, LC3-interacting region; MOI, multiplicity of infection; MS, mass spectrometry; PFU, plaque-forming units; PV, poliovirus; Rap, rapamycin; RHV-1a, Rhinovirus 1a; WT, wild-type. (TIF) [file pbio.2006926.s004.tif]

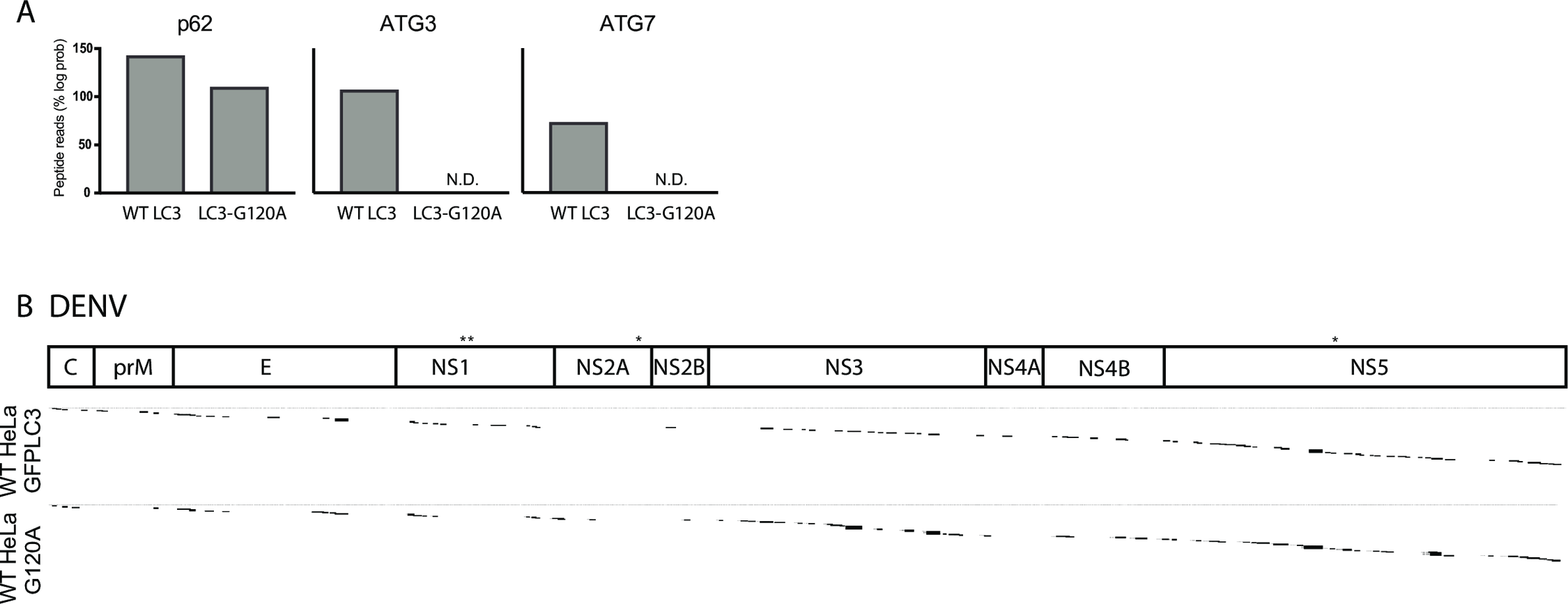

Supplement: S5 Fig — DENV proteins bind LC3. (A) BHK21 cells were transfected with GFP–LC3 or GFP–LC3–G120A for 48 hours and infected with DENV (MOI 10 PFU/cell) for 24 hours. Cells were lysed with buffer containing NP-40 and a GFP IP performed. Eluent was submitted for mass spectrometry and binding capacity assessed by comparing the log probability of peptide reads from infected samples to uninfected control samples, which were set to 100%. N.D. indicates no peptide reads were detected. (B) DENV peptide reads from the GFP–LC3 IP-MS were aligned to the DENV genome from LC3 and LC3–G120A samples. Stars indicate the location of WxxL LIR motifs in the DENV genome. BHK, bovine hamster kidney; DENV, dengue virus; GFP, green fluorescent protein; IP, immunoprecipitated; LC3, light-chain 3; LIR, LC3-interacting region; MOI, multiplicity of infection; MS, mass spectrometry; PFU, plaque-forming units. (TIF) [file pbio.2006926.s005.tif]
