# Supplementary material for: Differential and convergent utilization of autophagy components by positive-strand RNA viruses
Source: PLoS Biol. 2019 Jan 4;17(1):e2006926. doi: 10.1371/journal.pbio.2006926 (PMC6334974; doi:10.1371/journal.pbio.2006926)
Supplement: S1 Table — Sequences for guide RNAs. Nucleotides in bold denote the added cut site for the BBS1 restriction endonuclease site. (DOCX) [file pbio.2006926.s006.docx]

**Table S1: Sequences for Guide RNAs.** Nucleotides in bold denote the added Bbs1 cut site.

| Gene | Exon # | Guide sequence top | Guide sequence bottom |
| --- | --- | --- | --- |
| ULK1 | 1 | **CACCG**TCGCGGTGGTCTTCAAGGGC | **AAAC**GCCCTTGAAGACCACCGCGA**C** |
| FIP200 | 2 | **CACCG**CAAGATTGCTATTCAACACC | **AAAC**GGTGTTGAATAGCAATCTTG**C** |
| ATG9 | 1 | **CACCG**CTGTTGGTGCACGTCGCCGA | **AAAC**TCGGCGACGTGCACCAACAG**C** |
| BECN1 | 2 | **CACC**GGCGAAACCAGGAGAGACCC | **AAAC**GGGTCTCTCCTGGTTTCGCC |
| VPS34 | 3 | **CACC**GGAACAACGGTTTCGCTCTT | **AAAC**AAGAGCGAAACCGTTGTTCC |
| LC3B | 1 | **CACCG**TTCAAGCAGCGCCGCACCTT | **AAAC**AAGGTGCGGCGCTGCTTGAA**C** |
| ATG5 | 2 | **CACCG**AAGAGTAAGTTATTTGACGT | **AAAC**ACGTCAAATAACTTACTCTT**C** |
