# Supplementary material for: Differential and convergent utilization of autophagy components by positive-strand RNA viruses
Source: PLoS Biol. 2019 Jan 4;17(1):e2006926. doi: 10.1371/journal.pbio.2006926 (PMC6334974; doi:10.1371/journal.pbio.2006926)
Supplement: S2 Table — Primer sequences for quantitative PCR. Primers used for viral genomic RNA and for the murine ATG5 gene that contained the flox sites required for CRE-specific clevage. (DOCX) [file pbio.2006926.s007.docx]

**Table S2: Related to Figure 2 and Figure S1. Primer sequences for qPCR.** Primers used for mAtg5 floxed gene and viral genomic RNA.

| Gene | Name | Forward | Reverse |
| --- | --- | --- | --- |
| mAtg5 | Primer set 1 | GTACTGCATAATGGTTTAACTCTTGC | CAGGGAATGGTGTCTCCCAC |
| mAtg5 | Primer set 2 | ATATGAAGGCACACCCCTGA | CAGGGAATGGTGTCTCCCAC |
| 2A | Poliovirus | GTAGAGACCTCTTAGTCACAGAATCAAGAG | ATGGCCAATGAGCATATGGGACATG |
| NS3A | DENV | AATGGGTCTCGGGAAAGGAT | AAGAGCTGCTGTGAGAGTTA |
| NS2A | ZIKV | CCGCTGCCCAACACAAG | CCACTAACGTTCTTTTGCAGACAT |
